# Peanuts (*Arachis hypogaea* L.) and Mycotoxins: Analytical Approaches, Prevalence, and Innovative Detoxification ^†^

**DOI:** 10.3390/foods14050902

**Published:** 2025-03-06

**Authors:** Beatriz Melo, João Robalo, Fernando Ramos, Ana Sanches Silva

**Affiliations:** 1 University of Coimbra, Faculty of Pharmacy, Azinhaga de Santa Comba, 3000-548 Coimbra, Portugal; 2 Associated Laboratory for Green Chemistry (LAQV) of the Network of Chemistry and Technology (REQUIMTE) (REQUIMTE/LAQV), R. D. Manuel II, Apartado 55142, 4051-401 Porto, Portugal; 3 Center for Study in Animal Science (CECA), Instituto de Ciências, Tecnologias e Agroambiente (ICETA), University of Porto, 4099-002 Porto, Portugal; 4 Associate Laboratory for Animal and Veterinary Sciences (Al4AnimalS), 1300-477 Lisbon, Portugal

**Keywords:** aflatoxins, co-occurrence, chromatography, decontamination, dried fruits, groundnuts, immunoassay, multi-analyte, mycotoxins, nuts

## Abstract

Mycotoxins are secondary metabolites originating from several species of fungi that have proven to demonstrate high toxicity. In addition, potential contamination sources can promote increased human exposure to the adverse effects of these toxins. For this reason, it was necessary to develop several analytical methods that allow detection with the highest possible sensitivity for these toxic metabolites. Furthermore, since these methods involve high cost, are lengthy, and have sensitivity requirements, the development of multi-analyte detection methods is indispensable. The increasing consumption of groundnuts (legumes) as well as nuts (such as almonds, walnuts, and pistachios) and dried fruit (raisins and dried figs) has increased the risk of poisoning and the harmful effects of mycotoxins, which has encouraged studies for the creation of these methods. This review addresses the most representative methods applied to analyze and quantify mycotoxins in groundnuts (peanuts) together with decontamination techniques. The methodologies presented in this review are primarily based on analytical techniques for nuts and dried fruits. However, each of these methodologies can also be applied to peanut analysis for comparison and use. It is also relevant to highlight the importance of the development of multi-analyte methods in order to identify multiple mycotoxins using a single method, saving time, costs, and resources.

## 1. Introduction

Peanuts (*Arachis hypogaea* L.), also known as groundnuts in some regions of the world, are the edible seeds of a legume that can grow in the tropics, subtropics, and warm temperate climates [1,2]. Its scientific designation, given by Carl Linnaeus, ‘Arachis’ means ‘legume’ and ‘Hypogaea’ refers to the production of pods in the soil and means ‘under the earth’ [2]. Peanuts are classified as peas and are part of the bean/legume family (Fabaceae). Additionally, this legume is a good source of plant-based protein, unsaturated fat, oil, and fiber (complex carbohydrate) [1,2].

In addition to their rich nutrient profile, according to the literature, peanuts can be considered a functional food, with several health benefits. Besides being a good source of dietary fiber and other nutrients, as mentioned before, peanuts provide several bioactive components, such as Vitamins B and E, antioxidants (resveratrol and phenolic acids), and a wide range of minerals such as iron, zinc, potassium, magnesium, selenium, manganese, and copper [2]. These bioactive components have been shown to have disease-fighting capabilities, reducing the risk of diabetes, gallbladder disease, cancer growth, and protecting against Alzheimer’s disease. Furthermore, the consumption of peanuts and of some peanut-based products have been shown to help to mitigate malnourishment and improve weight management, reducing hunger and body mass index values [1].

Despite all the health benefits of peanuts, there are scientific evidence of their susceptibility, during storage and transportation, to contamination by fungi, particularly *Aspergillus flavus*, a fungal species that produces a highly toxic variety of mycotoxins named aflatoxins [1]. In addition to jeopardizing food safety, the infection of peanuts with these highly toxic mycotoxins is also a big concern in the worldwide trade of groundnuts, severely impacting the export market of developing countries [2].

Mycotoxins, such as aflatoxins, are secondary metabolites produced by fungi, mainly *Fusarium* spp., *Aspergillus* spp. (e.g., Aspergillus *flavus* and *Aspergillus paraticus*), and Penicillium spp., which have the potential to cause a wide spectrum of toxicological effects on human health. Mycotoxins can be formed under specific conditions, such as high temperature or humidity, on a variety of substrates, as well as in the presence of enormous amounts of nutrients. Due to its characteristics, many countries, especially developing countries, consider them to be among the most concerning food contaminants [3,4].

Nuts, a group of oleaginous fruits consumed in the dried form, in which peanuts are commonly included despite being technically legumes, are increasingly popular and more widely used in the European diet. However, their intrinsic moisture and nutrient content, long shelf life, high pH, and water activity are all factors that boost increased fungal growth; therefore, it is necessary to pay attention to the susceptibility of these foods to contamination by fungi [3,4]. Peanuts are among the most produced nuts worldwide, surpassed only by almonds, walnuts, and cashews [5]. In developing countries, peanuts play an important role as an oil crop and food crop, whether it is for domestic consumption or to export [2]. According to FAOSTAT (Food and Agriculture Organization Corporate Statistical Database) [5], the major producers of shelled groundnuts in 2020 were China, India, and Nigeria, and they have been maintaining this record from 2000 to 2020. Especially in developing countries, the production is restricted by several factors, such as farming in marginal areas under rainfed conditions, the occurrence of repeated drought stress due to monsoon vagaries, and a greater incidence of pest attacks, disease, low input farming systems, and socio-economic constraints [2,6].

Food contamination by mycotoxins is a pressing issue on a global scale. Even with good agricultural practices, storage, and processing, mycotoxin contamination is seen as an inherent and unpredictable problem, being a difficult challenge to food safety, especially in developing countries [7]. In the European Union (EU), there is a rising concern about the effects of mycotoxins on human and animal health. In this line, Commission Regulation (EC) 1881/2006 [8] and the respective amendments establish maximum permitted levels for certain mycotoxins in groundnuts, nuts, and dried fruits, such as aflatoxin B1 (AFB1), the sum of aflatoxins B1, B2, G1, and G2 (AFBs), and Ochratoxin A (OTA).

Mycotoxin contamination, particularly in aflatoxins, poses significant economic and social challenges for peanut farming communities, yet these aspects are often overlooked in scientific reviews. Economically, aflatoxin contamination leads to severe financial losses due to rejected exports, reduced market value, and increased costs associated with testing and mitigation strategies. Smallholder farmers, who often lack the resources for proper storage and monitoring, are disproportionately affected, facing income instability and potential livelihood loss. Socially, mycotoxin exposure has serious health implications, including chronic toxicity and an increased risk of liver cancer, which can strain healthcare systems and reduce workforce productivity. Additionally, consumer trust in peanut-derived products may decline, further exacerbating economic hardships. Addressing these broader impacts is crucial for developing comprehensive solutions that integrate scientific, economic, and social perspectives, ensuring the sustainability of peanut farming communities while enhancing food safety [7]. Worldwide, massive efforts have been undertaken to prevent or reduce the presence of mycotoxin in food, including peanuts. In this sense, various accurate and precise analytical techniques have been developed to reduce the exposition of humans and animals to mycotoxin contamination [7]. The main purpose of this review is to identify which are the most common mycotoxins in groundnuts and their co-occurrences and compare them with those found in nuts and dried fruits. Moreover, the most relevant and suitable analytical methods to carry out a multi-mycotoxin analysis in groundnuts were systematically reviewed and also compared with those applied to nuts and dried fruits. Finally, mycotoxin detoxification processes applied to groundnuts were also addressed and discussed.

## 2. Mycotoxins

Mycotoxins are secondary metabolites produced by fungi that, if ingested, can cause adverse effects not only on humans but also on animal health. They are regarded as one of the most significant food contaminants in various regions of the world, which has led to a wide range of risk assessment research studies [4,9].

As mentioned earlier, the susceptibility to mycotoxin contamination depends not only on the inherent properties of food (such as nutrient content, pH, and water activity), but also on storage conditions (e.g., high humidity and storage time) [9].

There are several types of mycotoxins, namely aflatoxins, OTA, patulin, deoxynivalenol (DON), zearalenone (ZEA), fumonisins (FB1, FB2), and T-2 and HT-2 T toxins [7]. However, this review focuses on the mycotoxins that most frequently affect peanuts, nuts, and dried fruits. In this line, aflatoxins are specially addressed due to the fact that the Commission Regulation no 1881/2006 [8] and its amendments established maximum permitted values for these mycotoxins in groundnuts (peanuts), nuts, and dried fruits. Dried fruits also have maximum permitted levels for OTA. As it may be confirmed in this EU Regulation, mycotoxins’ maximum permitted values allowed for groundnuts (peanuts) are in the same range as other nuts; however, dried fruits have a lower maximum permitted value. Other mycotoxins are arising, so-called emerging mycotoxins. The emerging mycotoxins include enniatins (ENs) and beauvericin (BEA), which are cyclic hexadepsipeptides [4].

### 2.1. Aflatoxins

Aflatoxins are secondary metabolites produced by Aspergillus species, mainly *Aspergillus flavus* and *Aspergillus parasiticus*. They are groups of heterocyclic aromatic hydrocarbons, and their production is intensified under conditions of high temperature and humidity [10]. In addition, aflatoxins are the most prevalent mycotoxins in foods, cereals, milk, and oilseeds [11]. Due to their high hepatocarcinogenic potential, aflatoxins are recognized by International Agency for Research on Cancer (IARC) as a Group 1 human carcinogen [12,13,14].

Aflatoxins B1 (AFB1), B2 (AFB2), G1 (AFG1), and G2 (AFG2) have been characterized under UV light out of over 20 types of aflatoxins that have been discovered so far. AFB1 and AFB2 show a strong blue fluorescence, whereas AFG1 and AFG2 exhibit greenish-yellow fluorescence under UV radiation, hence the designation of B (blue) and G (green) [15,16]. Additionally, the evidence suggests a difference in the origin of aflatoxins, since only AFB1/AFB2 are produced by *Aspergillus flavus,* whereas AFB1/AFB2/AFG1/AFG2 are produced by *Aspergillus parasiticus* [15,17].

AFB1 is considered one of the most toxic mycotoxins, as well as one of the most harmful to human health. The mean lethal dose (LD50) of AFB1 is 0.36 mg/kg (body weight) [10] and is classified as a group 1 carcinogen by the international agency of research on cancer (IARC) [15]. Moreover, it is considered to be the most potent hepatocarcinogen [14,18].

Aflatoxins, including AFB1, AFB2, AFG1, and AFG2, can take place in a wide range of raw materials, especially on foods stored in warm and moist conditions such as peanuts. To avoid human exposure to aflatoxins, it became imperative to develop fast and sensitive methods to analyze agro-products [3,14].

Among aflatoxins, aflatoxin M1 (AFM1) is the principal hydroxylated metabolite of AFB1, produced by the action of cytochrome P450 1A2 (CYP1A2) [19,20,21]. It is strongly fluorescent with the emission of blue-violet light. This toxin can be found in milk produced by mammals that consumed contaminated feedstuff [19].

The aforementioned aflatoxins were reported to have hepatotoxicity, mutagenicity, carcinogenicity, immunosuppression, neurotoxicity, epigenetic effects, reproductive dysfunction, and stunted growth [15]. Besides their potentially harmful effects on the human body, potentially leading to liver cancer, in animals, they can lead to weight loss and reproductive issues [9,21]. Particularly, in swine, they can cause acute hepatitis, systemic hemorrhages, nephrosis, and death, in addition to lowered stress tolerance [22]. Given this high toxicity, it is of utmost importance to regulate the maximum permitted values at the global level, and, in this regard, the development and validation (according to the international guideline) of analytical methods suitable to accurately determine the mycotoxins’ contamination levels of foodstuffs are imperative.

### 2.2. Ochratoxin A

OTA, similar to aflatoxins, is a secondary metabolite produced by fungi of the *Aspergillus ochraceus* and *Penicillium verrucosum* species [23] and, according to the IARC, is classified and listed in group 2B of the list of carcinogens [12,24].

Due to the possible genotoxic character, several in vivo and in vitro studies have been performed and shown that guanine- and OTA-specific DNA adducts persisted for more than 16 days and 5 days, respectively, in situations of genotoxicity at the hepatic and renal levels [11].

It is possible to obtain phase I and phase II OTA metabolites if they are hydrolyzed. This chemical reaction can occur in the stomach by proteolytic enzymes, being transformed into α-ochratoxin, but there is also the possibility that they undergo hydrolysis in the intestine since their alkaline pH causes the lactone ring to open, causing very high toxicity. The plurality of OTA metabolites from phase I and phase II detoxification present low toxicity, and, due to their strong binding to albumin, their elimination through glomerular filtration is insignificant, their excretion mainly occurring through tubular secretion; for this reason, it is the site where a more intracellular accumulation of OTA occurs [11]. In addition to accumulation at the tubular level, OTA can also accumulate in the liver and muscles due to their high affinity for proteins and their non-ionized form, which allows them to remain longer in the body. The kidneys are one of the greatest organs for the storage of these mycotoxins as they reabsorb OTA in the proximal and distal tubule, which contributes to the high level of nephrotoxicity [11].

### 2.3. Fumonisins (FB1 and FB2)

Fumonisins are found especially in maize and cereals and are derived from products produced by *Fusarium proliferatum* and *Fusarium verticillioides*. There exist 28 compounds, the most frequent being isfumonisin B1 (FB1) [25,26]. Additionally, FB1, as well as FB2, is listed as a potential carcinogenic substance in group 2B of the IARC [26].

These toxins are considered to be carcinogenic, hepatotoxic, immunotoxic, nephrotoxic, and neurotoxic, possibly provoking liver damage, heart failure, and esophageal cancer. They were found in several food supplements, such as green coffee, milk thistle, mint, chamomile flower, and liquorice [26].

### 2.4. Zearalenone (ZEA)

ZEA is produced by the genus Fusarium, particularly by *Fusarium graminearum* and *Fusarium culmorum,* and they are very common in cereals, such as maize and derivates [7]. It is classified as group 3 in the IARC, not being considered carcinogenic to humans [12].

ZEA has an affinity for estrogen receptors because of its structural similarity to 17-β-estradiol, a sex hormone, and may lead to adverse impacts on the reproductive system of humans and cattle [26].

### 2.5. Trichothecenes

Trichothecenes are secondary metabolites produced predominantly by Fusarium. This group of mycotoxins includes DON, HT-2 toxin, and T-2 toxin. They may cause skin irritation, induce problems in the stomach and intestine, and disturb mitochondria functions and the hypothrophia of spleen and thymus [26]. Both T-2 toxin and DON are classified as group 3 according to the IARC [12].

DON is essentially produced by *Fusarium graminearum* and *Fusarium culmorum* (Ahmad). It is common in cereals and cereals products [12]. It is known to be responsible for nausea, vomiting, abdominal pain, headache, and fever due to its vomitoxin action [27]. Concerning HT-2 toxin and T-2 toxin, these mycotoxins are mainly found in cereals [12] and are produced by *Fusarium sporotrichioides* and *Fusarium poae* [26]. T-2 toxin has a hematotoxic effect and is connected to alimentary toxic aleukia (ATA), which causes gastrointestinal irritation, vomiting, and diarrhea [28].

### 2.6. Emerging Mycotoxins

There are also some new mycotoxins arising, the so-called emerging mycotoxins. Emerging mycotoxins are gaining attention due to their increasing prevalence in food and feed, driven by changing agricultural practices and climate conditions. Climate change, with rising temperatures and altered humidity levels, may further enhance fungal growth and mycotoxin production, potentially leading to new contamination challenges.

Unlike well-known mycotoxins, emerging mycotoxins are not yet fully regulated, and their toxicological effects are still being explored. The emerging mycotoxins include enniatins (ENs) and beauvericin (BEA) and are predominantly derived from Fusarium fungi [4]. They are cyclic hexadepsipeptides with insecticidal properties capable of inducing apoptosis in mammalian cells. In addition, they have antibiotic properties against Gram-positive bacteria and mycobacteria [4,29]. They can cause a wide range of toxic effects in humans and animals, which could lead to the development of carcinogenic, teratogenic, and mutagenic effects, affecting the production of hormones and immunosuppression [4,30].

They act as enzyme inhibitors, antibacterial and antifungal agents, and immunomodulatory substances. They have the capacity to inhibit the enzyme acyl-CoA and cholesterol acyltransferase.

### 2.7. Co-Occurrence of Mycotoxins

This phenomenon has been reported in different food samples, and it consists of the occurrence of several mycotoxins in the same matrix. A food item is frequently more exposed to multiple mycotoxins than to a single mycotoxin; for this reason, it is expected to produce combined effects due to the association of the different mycotoxins [31]. The actual risks to health have yet to be studied due to the lack of information available on potential effects [31,32].

Frequently, one sample is contaminated with more than one mycotoxin, and the most reported cases include the co-occurrence of AFB1 and AFB2. In a recent study, a peanut paste sample was described to be the most contaminated sample with multi-mycotoxins, essentially by AFB1 and total aflatoxins (AFB1, AFB2, AFG1, and AFG2), as well as different fungi [33].

## 3. Toxicity Mechanisms of Mycotoxins

Cytochrome P450 enzymes (CYP450) are present in the organs where mycotoxins present greater toxicity, such as liver, intestines, and kidneys. These enzymes are responsible for biotransformation by the oxidation of organic extracts, but also for the biotransformation of xenobiotics, leading to their oxidation [11,34]. The CYP450 action is dependent on the inhibitory, inductive, or substrate capacity of the mycotoxins, which consequently determines the changes that mycotoxins metabolically cause. It is possible that the action of mycotoxins can alter the absorption and biotransformation of nutrients and other substrates that may be present in organisms [11].

Popescu et al. [11] analyzed piglet livers and kidneys and found differences enhanced by exposure to OTA and AFB1, showing an alteration of the gene expression on the type of gene. In this study, piglets were divided into three groups. The first one (E1) was fed with a basal diet plus a mixture (blend) of two byproducts (grapeseed and sea buckthorn meal); the second group (E2) was fed with a basal diet experimentally contaminated with mycotoxins AFB1 and OTA; and the third group (E3) was fed with a basal diet containing 5% of the previously mentioned mixture of the byproducts and contaminated with the mix of AFB1 and OTA. In piglets contaminated with OTA and AFB1, the inclusion of grapeseed and sea buckthorn (E3) decreased the expression of the CYP P450 gene. The decreased expression of this gene induces the decreased bioactivation of the mycotoxins, possibly resulting in decreased toxicity in both organs under study [11]. Therefore, both grapeseed and sea buckthorn can be considered sources of the neutralization of the harmful effects of OTA and AFB1. However, the author concluded that further tests are needed to understand which derived products with antioxidant action decrease or increase the expression of CYPs mRNA and how this process occurs [11]. In other aspects, the light microscopic analysis of the liver from a group of piglets fed with a basal diet contaminated with a mixture of AFB1 and OTA exhibited local areas with necrosis, the dilatation of sinusoid, and inflammatory parenchymal infiltration. Mononuclear cellular infiltration and periportal fibrosis were revealed, as well as fibrotic perilobular and fibrotic septa [11]. The analysis of the structural changes in kidneys demonstrated that the administration of mycotoxins AFB1 and OTA affected both the medulla and cortex. The atrophy of the glomerular tufts and the alteration of the Bowman’s capsule were noticed, as well as the tubular necrosis of lining epithelial cells with inflammatory cell infiltration in between. Also, between the glomeruli and tubules, focal aggregates of inflammatory cells were observed to be in association with focal areas of congestion in blood vessels, especially in the medulla. In this study, collagen proliferation occurred in areas with tubular injury. The kidney section from the group fed with a basal diet with the mixture of by-products and contaminated with a mixture of AFB1 and OTA manifested minor pathomorphological changes, similarly to the control [11]. Concerning the level of gene expression, the conclusions that can be drawn from this study indicate the concomitant administration of the mixture of grapeseed and sea buckthorn meal, and AFB1 and OTA promoted a decrease in all analyzed gene expressions in the liver compared to the control group.

## 4. Mycotoxins Contamination

### 4.1. Preharvest Contamination Sources

There are different sources of food contamination by mycotoxins depending on the soil, use of pesticides, animals, harvest, handling, and storage [35]. Firstly, soil type, humidity, surface and subsurface temperature, and air temperature are characteristics that influence fungi and mycotoxins [35]. Repetitive mowing, use of herbicides and other pesticides, and grazing using animals or not are aspects that have to be considered to control the spread of these toxins [35,36]. Results in some studies indicate that contaminations in peanuts were higher when soils were grazed compared to ungrazed soils, with higher contaminations found in the samples from the wet year compared to the samples from the dry year. Also, when the peanut shells are intact, fungi can not penetrate to the inside of in-shell peanuts [35,37]. Water is used for several practices in the soil, whether for irrigation, mixing pesticides, or fertilizers, so it is necessary to ensure that the water is safe and has adequate quality for use [35].

Secondly, if dried fruits, nuts, or legumes are harvested from the ground, soil must be prepared prior to harvesting in order to ensure proper safety and no potential contamination, such as animal feces, uncleaned equipment, and non-trained workers. For this situation, all workers must be trained regarding good health and hygiene practices in harvesting activities, and the equipment must be correctly cleaned. Mechanical harvesting should be processed in dry weather in order to minimize potential cross-contamination [35].

The handling process is another important step. Some nuts are mechanically dried after harvesting in order to reduce the moisture content, which is critical to prevent microbial growth that may contribute to mycotoxins, namely aflatoxin formation. The reduction in pathogens on the surface of dried fruits, nuts, or peanuts depends on several factors. The time, temperature, type of heat treatment, type of organisms, and type of food items are all critical parameters to assure the lethality of mycotoxins. The use of heat treatments is able to ensure adequate and significant reductions in mycotoxins [35].

After all these steps, the storage should occur in a facility that is dry and guarantees protection. It should have minimum temperature fluctuations to prevent microbial growth and insect infestation. Therefore, the storage of the products is essential under controlled conditions to maintain their quality and their shelf life [35,38].

### 4.2. Climate Changes and Mycotoxins

For a complete understanding of the toxin contaminations and their results, it is important to have an overview of the environmental factors affecting fungi survival, growth, interactions, and metabolic activity [39,40].

The environment may provide all the factors to enhance mycotoxin prevalence; particularly, high temperatures and droughts affect crops, like maize, and also the occurrence of *A. flavus*, boosting fungal growth [39,41]. Therefore, climate change is predicted to have a significant impact on mycotoxins. According to certain available data, concentrations of CO_2_ are projected to double or triple in actual concentration in the next 25 to 50 years. The temperatures of different regions in Europe are expected to increase by 2 to 5 °C due to the concentrations of CO_2_ and drought episodes [39,42,43,44]. Because of this situation, the European Food Safety Authority (EFSA) requested the identification of emerging risks and has identified changing patterns in mycotoxin production [39], and, for this, they funded the project MODMAP-AFLA. This provided data that specifically analyzed maize so that it was possible to predict subsequent aflatoxin contamination risks. The project predicted an increased risk of aflatoxin contamination in the future [39,45].

The data collected also suggested that climate change effects depend on geographical regions [39,46]. Mediterranean regions are expected to be affected by many adverse effects, impacting aflatoxin contamination [39].

The essential topic for defining the impact of fungal co-occurrence under the influence of different meteorological and ecological conditions on mycotoxin contamination [39,47,48] was studied in the field and in vitro [39], where fungal interactions could be exhibited under natural conditions, except for temperature and water activity (aw). Under the influence of different temperatures, the production rate dynamic decreased and the optimal temperature for AFB1 suffered some alterations [39]. During these studies, the environmental and toxicological consequences of aflatoxins contamination were considered, clarifying the eventual risks that aflatoxin contamination represents in soil ecosystems [39,47]. Climatic strategies play a crucial role in future risk assessments of aflatoxin contamination, as climate factors such as soil moisture and air temperature significantly influence fungal growth and toxin production [39].

Another study was carried out to examine different climate change scenarios, considering a 2.5 times increase in CO_2_ in southern Italy [39,48]. In consideration of the rise in concentration, preliminary evidence indicated that temperature increases may reduce the OTA productions [39,49]. With a particular temperature range of 18/31 °C and particular water stress conditions (0.93 aw), it was observed that the fungi growth rate was slower than 0.99 aw, and an over-expression of OTA genes was also observed. In the opposite situation, with a temperature range of 20/37 °C, a higher growth rate of 0.93 aw was exhibited. Consequently, higher temperatures and water stress appear not to be favorable for OTA production, and this situation must be confirmed in the future [39].

On a global level and based on the predictions of the AFLA-maize model, AFB1 is expected to increase as a result of climate change. In this way, predictive models have been shown to be crucial for addressing future uncertainties and anticipating risk conditions [39].

The rise of the risk associated with aflatoxins is related to fungi and mycotoxin co-occurrence; for this reason, in future studies, it is important to interpret and convert results through the careful evaluation of developing countries in order to predict the impact of climate changes on aflatoxins and other mycotoxin occurrences [39].

### 4.3. Mycotoxins Legal Status at EU Level

The Commission Regulation (EU) no 1881/2006 [8] and its amendments establish the maximum levels for certain contaminants, namely some mycotoxins, in the EU in foodstuff, namely in groundnuts, nuts, and dried fruits. According to this regulation, the maximum level for AFB1 is 8 µg/kg, while for the sum of B1, B2, G1, and G2, it is 15 µg/kg for groundnuts (peanuts) and other oilseeds to be subjected to sorting or other physical treatment before human consumption or use as an ingredient in foodstuffs, with the exception of groundnuts (peanuts) and other oilseeds used for crushing for refined vegetable oil production.

## 5. Determination of Mycotoxins

For the correct detection and determination of mycotoxins, three essential steps must be fulfilled: sample selection, sample treatment, and mycotoxin detection and quantification. It is crucial to comply with all the steps so that the sample is representative and treated correctly and so that the sample determination is specific, sensitive, and accurate. The various procedures will be described in the following sections. Each sequential step contributes to the variability of the method and avoids possible errors arising from the whole procedure.

### 5.1. Sampling and Pre-Treatment

Sampling is the selection of representative samples of the population to be analyzed. This selection is also important to circumvent difficulties of heterogeneous distribution, inducing higher precision when determining mycotoxins levels [50]. Commission Regulation (EC) no 401/2006 [51] lays down the methods of sampling and analysis for the official control of the levels of mycotoxins in foodstuff.

Depending on each lot’s weight, groundnuts, pistachios, Brazil nuts, and other nuts can be subdivided into sublots (≥500 t; >125 and <500 t; ≥15 and ≤125 t; <15 t), and each sublot has specific conditions [51]. Sample pre-treatment aims to reduce and, if possible, eliminate matrix effects; this means that it may ensure that components present in the sample will not interfere with the analyte’s quantification. Thus, mycotoxins must be extracted efficiently. In addition to extraction, further purification might be carried out to ensure that the sample is analyzed under optimal analytical conditions. On the other hand, if non-destructive techniques such as infrared spectroscopy or other optical methods are used, sample pre-treatment is not necessarily required, as these techniques allow for direct analysis without altering the sample’s composition [50].

### 5.2. Extraction and Purification

Due to the known adverse effects of aflatoxins, such as carcinogenicity, mutagenicity, and teratogenicity, the handling of working solutions, standards, and extracts should be performed with special care, in a fume hood while wearing the usual laboratory protections, such as lab coat, mask, gloves, and eye protection. Standard solutions should be kept away and protected from light in amber containers [50]. Organic solvents, like methanol, chloroform, and acetonitrile, are necessary for the extraction of aflatoxins from a variety of food matrices, and methanol/water and acetonitrile/water mixtures are commonly used for aflatoxin extraction [50]. Due to its potential ecological risk, the use of chloroform for aflatoxin extraction has been declining [50]. There are several methods for the purification of the target analytes. The correct purification of the sample determines the sensitivity of the analysis for detection [50].

#### 5.2.1. Liquid–Liquid Extraction (LLE)

Since aflatoxins are more soluble in polar organic solvents, such as chloroform, methanol, and acetonitrile, LLE is regularly used to extract and separate aflatoxins from solid food matrices. Although it is simple and does not require specialized work, the greatest disadvantage of this extraction technique is that it frequently involves a time-consuming procedure that uses a lot of organic solvent, particularly when processing many sample extractions [52]. Generally, when determining mycotoxin, samples are extracted, followed by evaporation in order to reduce volume and, consequently, concentrate mycotoxins in the extract. The mycotoxins present in the polar phase may be determined by thin-layer chromatography (TLC), and, subsequently, their identification and quantification can be obtained by comparisons of the retention factor (Rf) and respective intensity of the fluorescence of the samples, with standard TLC plates [52,53]. Dispersive Liquid–Liquid Microextraction (DLLME) emerged to improve the extraction process and to minimize solvent consumption and increase concentration factors [52,54]. This method allows us to preconcentrate target aflatoxins with a microvolume of the extraction phase [52]. DLLME uses the dispersion of the extracting solvent with a second solvent (dispersion solvent), which has to be soluble in water, to extract the analytes at hand [54].

#### 5.2.2. Solid-Phase Extraction (SPE)

SPE uses the selective retention of analytes at the same time to eliminate extract interferences, provided by the use of adsorbents with a strong affinity to the target analyte(s). Once there is a wide range of adsorbents, this method gained selectivity and flexibility, and the SPE cartridges contain predominantly silica gel, Florisil, C18, or polymeric adsorbents. It is important to pay attention to certain key parameters, for example, the type of adsorbent, elution solvents, and dilution factors [52,55]. In order to provide more sensitivity and selectivity, a procedure appeared using aluminum oxide as the adsorbent and methanol/water (80:20 *v*/*v*) as the extraction solvent, which retained compounds with high polarity in polar solvents and removed low polarity and non-polar interferents simultaneously from the extract, promoting the purification of aflatoxins prior to the analysis [52,55]. Matrix Solid-Phase Dispersion (MSPD) has been developed to simplify SPE, since it does not need cartridges to mix the sample and adsorbent, mixing them directly. The elution of the target analytes is then performed on the mixture after it has been packed into a glass column [52,56]. A study for the MSPD procedure has been evaluated in peanuts using C18 bonded silica as the dispersive adsorbent for the analysis of AFB1, AFB2, AFG1, and AFG2, applying acetonitrile as the eluting solvent followed by evaporation and LC-FLD to quantify [52,57]. Magnetic Solid-Phase Extraction (MSPE) is a technique that uses superparamagnetic nanoparticles, such as Fe_3_O_4_, as an adsorbent. Using an external magnet, the adsorbent can be separated by magnetic decantation, demonstrating its selectivity and its efficiency; however, it requires time, cost, and specialized professionals [52]. Another area worth developing is Solid-Phase Micro-Extraction (SPME), which was initially coupled with Gas Chromatography (GC), but recently was used with LC analysis for semi and non-volatile compounds [52,58]. It is not recurrently used due to the cost and limited selection of stationary phases of SPME [52].

#### 5.2.3. Immunoaffinity Chromatography Columns (IACs)

IACs use the specificity and high affinity between antibodies and mycotoxins to achieve a clean-up procedure. This method provides a high specificity even in complex matrices using simplifying sample analysis [52,59,60,61]. Through the percolation of the extract down the column, mycotoxins bind with the antibodies, and, after the wash step, a solvent is used to disrupt these bindings. In recent studies, the multi-mycotoxin IAC has been coupled with LC-MS, providing screening for AFs, DON, OTA, Fumonisins, HT-2 Toxin, T-2 Toxin, and ZEA simultaneously. The disadvantages of this technique are the complexity and cost caused by the necessity of the immobilization of different types of antibodies [52].

#### 5.2.4. Supercritical Fluid Extraction (SFE)

SFE uses carbon dioxide (CO_2_), which permits the extraction of non-polar analytes [52,62] because CO_2_ has a low critical temperature and pressure, at 31 °C and 7.3 MPa, respectively [52,63]. The major advantages are the reduction in organic solvents, the easy extraction procedure through the release of pressure, and the possibility to connect with analytical instruments [52]. The rise in the applied pressure and temperature and the addition of modifiers promotes the solubility of mycotoxins in CO_2_. This technique is not implemented at a large scale because it requires costs, time, and complex operations [52].

#### 5.2.5. QuEChERS Extraction

The QuEChERS method requires two steps: (1) first, an extraction step that promotes an equilibrium between an aqueous and an organic layer (acetonitrile—MeCN) through partitioning via salting-out is required; (2) then, a dispersive solid-phase extraction (d-SPE), that includes a clean-up procedure, is performed. Besides using reduced amounts of sorbent and extract, the second step reduces the need for additional centrifugation, filtration, or precipitation [64,65,66]. The efficiency of the QuEChERS method is determined by the characteristics of the target analyte, matrix composition, laboratory equipment, and analytical procedures [64]. Over the years, the QuEChERS method has been improved and, consequently, nowadays, depending on the chemical nature of the substance we want to extract, the quantity of solvents and salts and their combinations can be optimized to improve the efficiency of this extraction method. Furthermore, it is important to remark that by reducing the matrix effect, increasing sensitivity, and recovering the target substances, the QuECHERS protocol can be optimized and, therefore, the extraction performance can be improved. This promising technique, besides being a simple and effective extraction procedure, uses organic solvents and little amounts of the sample, following green chemistry principles [64,67]. This procedure has been used to isolate a broad spectrum of mycotoxins, such as AFs, fumonisins, OTA, enniatins, BEA, trichothecenes, T-2 toxin, and H-2 toxin, and has been applied to several food matrices, simplifying sample analysis [64].

### 5.3. Separation, Detection, and Quantification of Mycotoxins

Accurate and reliable detection is essential to be able to monitor the different mycotoxins in a given food product, in this case, more specifically, being peanuts, nuts, and dried fruits. Several techniques have been developed for this purpose, including chromatographic methods, capillary electrophoresis (CE), immunological methods, and non-destructive techniques [50]. The detection of mycotoxins can be carried out by conventional strategies, but more sensitive and efficient methods are emerging.

Due to the toxicity of AFB1, the most potent aflatoxin, and the strict regulations that limit the total levels of aflatoxins in foodstuffs, it is very complex to develop simple, selective, and sensitive methods for aflatoxin detection. In this context, the use of nanomaterials has ‘opened a door’ for the development of fast, accurate, ultrasensitive, and practical methods. Due to their macroscopic tunneling effects, nanoparticles can be used to achieve higher sensitivity values, shorter detection times, and lower detection limits [10]. Given their size, shape, functionality, and surface area, nanoparticles (such as Au/Ag nanoparticles, carbon-based nanoparticles, magnetic nanoparticles, quantum dots, up-conversion nanoparticles, metal–organic frameworks, and nano-functional DNA intelligent hydrogels) can be used to develop different detection systems for AFB1, specifically immunoassays, biosensors, and sample pretreatment before AFB1 detection [10].

#### 5.3.1. Chromatographic Methods

Chromatography is based on the separation of a mixture of compounds depending on their different affinities for the mobile or stationary phase, which results in different movements in the column, leading to a possible separation. There are several analytic methods based on this technique, such as Thin Layer Chromatography (TLC), liquid chromatography (LC), liquid chromatography–mass spectrometry (LC-MS), gas chromatography (GC), and CE [50].

TLC is a fast and practical method that can be used for the qualitative, quantitative, and semi-quantitative determination of aflatoxins. However, TLC has, progressively, been replaced by other techniques, such as HPLC, based on the fact that it presents low selectivity and reproducibility [50]. For this reason, researchers developed an enhanced form of TLC, high-performance TLC (HPTLC), that provides higher separation efficiency and sensitivity [52]. Another enhanced form of TLC is the over-pressured layer chromatography (OPTCL). In this technique, the mobile phase is pushed through a thin column with a significantly larger cross-section than regular LC columns [50]. However, although it is less time-consuming and requires less mobile phase when compared to HPLC and it is more efficient than conventional TLC, it has, almost, only been used for research purposes so far, most likely due to the system’s complexity [50,52].

LC initially was developed because it was believed that it could surpass conventional TLC in speed, accuracy, and precision. Nevertheless, over the years, several ways were investigated to enhance the performance of this technique, such as coupling ultraviolet (UV) and fluorescence detectors with a normal phase LC. Since aflatoxins exhibit strong fluorescence (blue and green), it was found that fluorescence detectors (FLDs) were preferable compared to UV-Vis detectors, providing higher specificity and better detection. In previous studies, following LC separation, the aflatoxins were extracted from the mobile phase using a flow cell with a fluorometric detector loaded with silica gel particles. Using a silica gel particles solvent, the aflatoxins were then absorbed and measured, reducing quenching. This discovery allowed the LC-FLD to become a key technique in the analysis of aflatoxins in foods [52].

Furthermore, over the years, reversed-phase LC, due to its simplicity, started to gain popularity. Nevertheless, to improve sensitivity and resolution researchers started to chemically alter aflatoxins using trifluoroacetic acid or a halogen (bromine or iodine) [52]. Therefore, the LC-FLD method is an indispensable approach but is not suitable for multi-mycotoxin detection since other mycotoxins do not exhibit fluorescence.

Along with the advancements in technology, ultra-high performance liquid chromatography (UHPLC) emerged. With this method, rapid and efficient separations were possible thanks to the use of columns packed with submicron particles, making it a technique of choice to analyze mycotoxins in foods. UHPLC-FLD can determine aflatoxins, OTA, and ZEA simultaneously, so this technique is suitable for multi-mycotoxin analysis. Photochemical derivatization was tested in order to increase the signal intensity; however, bromine and iodine are not suited because OTA and ZEA are incapable of resisting the derivatization step [52].

LC-MS is able to determine multi-mycotoxins using LC separation and mass-to-charge (*m*/*z*) techniques, which use molecular weight to increase specific identification. This technique is specific, sensitive, and able to quantify mycotoxins in one analysis (chromatographic run). LC-MS can be customized with different MS analyzers, with the purpose being to promote detection capacity [52]. LC–Triple Quadrupole Mass Spectrometry (LC-QQQ-MS) developed to be highly sensitive for quantitative analysis, and according to some studies, the number of mycotoxins simultaneously detected in one analysis could be from 39 to 300. Considering economic interests, efforts are being made in order to improve the precision and accuracy of the LC-MS methods, and LC-FLD methods are being substituted by LC-MS methods [52,68].

For this goal, LC-high-resolution mass spectrometry (LC-HRMS) techniques were used, including Time-of-Flight (TOF) and an Quadrupole–Orbital Ion Trap (Q-Orbitrap). LC-HRMS techniques allow us to obtain high-resolution data in full scan mode, which can provide sufficient specificity and selectivity for quantification. Moreover, the data acquisition rate, data mining software, and data storage systems, which are supported by identification criteria guidance, allow these techniques to improve [52,68,69].

LC-Orbitrap MS is considered an advanced and efficient method for mycotoxin analysis, which could be used for a quantitative and qualitative approach using collected data. It combines target analysis with a non-targeted screening of mycotoxins, providing high-resolution data acquisition rates and accurate mass spectra libraries [52,68].

There exists another type of chromatography, gas chromatography (GC), which is based on the volatility of aflatoxins. This technique is preceded by capillary column separation on a fused capillary column with a nonpolar phase followed by detection using MS [52,70].

##### Liquid Chromatography–Tandem Mass Spectrometry

Liquid chromatography–tandem mass spectrometry (LC-MS/MS) or LC-QQ-MS is used frequently as a method for the quantitative and qualitative analysis of mycotoxins. Electrospray ionization (ESI) is a method compatible with most chromatography separation systems, but also its efficiency depends on several factors, namely the treatment and quality of the sample. The combination of these two methods makes them into a sensitive and selective analysis system, but for this to occur, it is necessary to optimize the factors that affect the retention time [24,71,72].

Single analyte procedures are time-consuming [24,73], and there is no access to the interaction with factors that may affect the ion source [24,74]. On the other hand, multivariant optimization procedures [75] allow the investigation of interactions between experimental variables and have proven to be effective when applied to LC-MS/MS [24]. In a study carried out by Alsharif et al. [24], the LC-MS/MS method was combined with the QuEChERS technique in order to determine multi-mycotoxins in 120 commercial food samples obtained from Malaysia. For this, both LC-MS/MS and QuEChERS performances were evaluated. The factors taken into account for the evaluation were linearity through linear regression, precision, and detection limit. LC-MS/MS evaluation was important to determine the accuracy of the quantification when changing variables. This technique demonstrated good sensitivity with a low limit of detection (LOD) and limit of quantification (LOQ) for a successful determination of multi-mycotoxins.

#### 5.3.2. Capillary Electrophoresis (CE)

CE is an electrokinetic separation technique that provides the effective separation of components without using organic solvents. It can offer rapid and simple analysis with high column efficiency [50]. In some studies, the potential of CE miniaturization is shown with the necessary tracing studies for its application in food and mycotoxins. When CE is combined with MS, the complexity of the technique is similar to LC-MS, but, as a well-established method, it is preferable to LC-MS-based technologies [52].

According to a study [76] on maize samples, it was possible to separate aflatoxins AFB1, AFB2, AFG1, and AFG2 using a capillary column and an on-column laser-based fluorescence detector.

#### 5.3.3. Immunoassays

Immunoassays are based on the affinity and specificity between the antigen and antibody, which is a highly specific new method, essential for mycotoxin determination. Immunochemical methods can be used for the quantification of the reaction by competitive binding. The specificity of the antibody and the existence of specific mycotoxins are both characteristics that influence the accuracy of the immunoassays for mycotoxins, namely for aflatoxins [77]. There are three types of immunochemical methods: radioimmunoassay (RIA), chemiluminescence assay, and Enzyme-Linked Immunosorbent Assay (ELISA).

Concerning RIA, the sample extracts with specific antibodies and a constant quantity of radiolabeled mycotoxin are incubated. Thereafter, free mycotoxin and bounded mycotoxin are separated, and the determination of the radioactivity in those fractions is initiated. Through the comparison between the standard curve and the sample radioactivity in the bound fractions and free fractions, it is possible to determine the mycotoxin concentration. On account of the cost, the labeling of mycotoxins with tritium, and the disposal of radioactive waste, it is not extensively used [77,78]. According to Chu [78], it was possible to analyze AFB1 and OTA with this method through radioactive determination in each fraction.

The ELISA technique has two versions relative to the formed bound: direct ELISA and indirect ELISA. The direct ELISA uses a specific antibody coated on a solid phase and incubated with the enzyme conjugate, forming an aflatoxin–enzyme conjugate. The quantity of the enzyme-bound form can be determined by incubation with a specific substrate solution. The color formed is measured through comparison with the standard mycotoxin or by a spectrophotometer. This technique depends on the competition for antibody-binding sites, so the free mycotoxin concentration is inversely related to antibody-bound enzyme conjugate [77,79]. The case of the indirect ELISA involves the protein–aflatoxin conjugate and a secondary antibody. The conjugate is coated onto the microtiter plate, and the sample or standard aflatoxin is inserted. The quantity of antibody bound is detected when a secondary antibody conjugated to alkaline phosphate is added, providing a colored product. By comparison with the standard curve, it is possible to determine the mycotoxin concentration [77].

Shadbad et al. [80] used immunoaffinity columns for aflatoxin determination using PBS buffer methanol in order to elute aflatoxins. It was possible to determine the total number of aflatoxins (AFB1, AFB2, AFG1, and AFG2).

##### Time-Resolved Fluorescence Immunochromatography Assay (TRFICA)

TRFICA is a new sensitive detection method that is considered to increase the sensitivity’s detection compared to the gold nanoparticle-based strip assay (GNP-SA). Using lanthanide chelate-embedded nanoparticles or microbeads with antibodies or proteins, TRFICA can be used for quantitative detection, as it has high sensitivity, a wide linear range, and low background. Since lanthanide chelates have a long fluorescence lifetime, wide excitation spectrum, sharp emission spectrum, and large Stokes shift, they demonstrate high-fluorescence characteristics. In order to increase the fluorescence intensity, lanthanide chelates were embedded into microbeads. This situation also provides the resolution of the limitation of the conventional dissociation-enhanced lanthanide fluorescence immunoassay [3]. Nitrocellulose membranes sprayed with test and control lines, used as a sample pad, and an absorbent pad are combined into a TRFICA device. The liquid sample present in the nitrocellulose membrane moves through capillary action to the absorbent pad, which provides the demonstration of the presence or absence of aflatoxins [3].

In the case of the absence of aflatoxins, the fluorescence lanthanide microbead-labeled antibodies create the aflatoxin–protein conjugate with the immobilized antigen on the test line and react with the secondary antibody on the control line. For this reason, it is possible to observe two colored lines under ultraviolet radiation [3].

On the other hand, when aflatoxin is present in the sample, the immobilized aflatoxin–protein conjugate competes with the aflatoxin present to possibly bind with the lanthanide microbead-labeled antibodies. This action is negatively correlated with the aflatoxin concentration in the sample [3].

This technique has been applied to different samples, such as maize and peanuts, to determine AFB1 and total aflatoxins (AFB1, AFB2, AFG1, and AFG2). The studies on the determination of AFB1 in food demonstrated rapid and effective results of the TRFICA technique [81]. Ovalbumin (OVA) is used to block the sample pad to prevent non-specific absorption. The test and control lines are covered with AFB1–bovine serum albumin (AFB1-BSA) and immunoglobulin G (IgG). For the analysis, it is necessary to first mix free AFB1 and monoclonal antibodies with AFB1 (anti-AFB1 mAb)-conjugated fluorescence microbeads (Eu3+) in a reaction pool, followed by lateral flow through TRFICA via capillary action. A binding interaction of aflatoxin–antiaflatoxin mAb and antiaflatoxin mAb-IgG is formed, and the detection results are consequently based on the fluorescence intensity of the test line and control line read by the TRFICA detector. This method demonstrates a better sensitivity compared to ELISA and GNP-SA methods [3].

For the determination of total aflatoxins, a coated AFB1-conjugated BSA and rabbit antimouse IgG are used on the nitrocellulose membrane for, respectively, the test line and control line. Homemade antiaflatoxin mAb was purified with a protein G immunoaffinity column. The rest of the method function is similar to the one described above. The binding interaction of aflatoxin–antiaflatoxin mAb and antiaflatoxin mAb-IgG induces fluorescence for detection. The detection sensitivity is higher compared to ELISA and GNP-SA methods [3].

#### 5.3.4. Aptamer-Based Biosensor

Most quantitative methods for the detection of mycotoxins mention TLC, HPLC, and LC-MS, but, in the meantime, aptamer-based biosensors have been developed and introduced as a new method for mycotoxin detection [23].

Aptamers are single-stranded (ss) DNA or RNA oligonucleotides, an alternative molecule to antibodies for recognition elements. The aptamers can form aptamer/target complexes with strong affinity and high specificity. Compared to antibody-based immunoassays, the aptamer-based biosensor uses smaller molecules, is screened and chemically synthesized in vitro, can be stored and transported at room temperature (less sensitive), is cheaper, requires a lower time of preparation, has no obvious immunogenicity, presents a wider range of target substances, keeps original biological activities with labels, and can separate structural analogs or cross-reactive substances, as well as making the temperature-induced denaturation reversible [23]; however, expensive and special instruments, a pre-treatment, and professional personnel are required for the use of this method.

There are several aptasensors developed for OTA analysis, such as fluorescent, colorimetric, and electrochemical aptasensors, as well as nanomaterial-based methods. A fluorescent aptasensor has been applied to the determination of mycotoxins in peanuts using the principle of aptamer-conjugated magnetic beads (MBs) and CdTe quantum dots (QDs). At the time of OTA addition, the production of the aptamer/OTA complex starts, and magnetic separation results in a fluorescence intensity enhancement. This one-step fluorescent aptasensor method provided wide-range responses and a lower limit of detection, which might represent a potential strategy for OTA control [23].

Since aflatoxins are the most toxic mycotoxins, according to the IARC classification, aptamer-based biosensors have been shown to have a high affinity to AFB1. For this reason, a variety of methods have been developed, such as fluorescent aptasensors, colorimetric aptasensors, Surface-Enhance Raman Scattering (SERS) aptasensors, electrochemical aptasensors, Microring Resonator aptasensors, electrochemiluminescence aptasensors, and Microcantilever array sensors. According to a study carried out by Li et al. [82], SERS is an analytical method able to be applied to large amounts of contaminated agricultural products. After AFB1 addition, the aptamer and AFB1 form the aptamer/AFB1 complex, leading to the dissociation of complementary DNA. While DNA suffers hybridization, the SERS tag was conjugated on the surface of AuNPs. Recently, SERS has been developed as an ultrasensitivity technique through CS-Fe_3_O_4_ nano-bead signal enrichment. When AFB1 is added, the SH-DNA2-ADANRs are induced to be released from the surface of CS-Fe_3_O_4_ as a result of the competitively binding reaction between AFB1 and NH_2_-DNA1-CS-Fe_3_O_4_, which leads to a decrease in the SERS’ signal. Both techniques have been successfully developed, but the ultrasensitive SERS aptasensor exhibits the advantages of the SERS technique, improving the detection stability and sensitivity [23].

This method has several advantages, such as high sensitivity and selectivity, rapid, portable, and low-cost target analysis, great potential for field determination, and the high-throughput identification of multiple mycotoxins [23].

Despite all the advantages, this technique uses small molecules; consequently, the screening of nucleic acid aptamers specific to mycotoxins is a complicated process and requires some time for this particular selection. In addition to this, naturally, the molecules have to be strongly specific to each mycotoxin. For this reason, few nucleic acid aptamers have been screened and applied successfully. Some mycotoxins are constituted with various structural analogs, so it is more difficult to select the highly specific aptamers for each analog. All in all, the greatest problem for this development is how to identify and detect multi-mycotoxins using a limited variety of mycotoxin aptamers, specifically for similar structure mycotoxins [23]. Apart from that, traditional antibody preparation needs animal experiments, which are difficult to prepare in large quantities; also, this preparation has technical barriers that could lead to its monopolization. Also, the affinity of aptamers is normally weaker than antibodies’ affinity. Finally, commercial kits have to consider diverse factors, for example stability, sensibility, cost, replicability, and technical barriers [23].

Another technique is a DNA-scaffolded silver nanocluster and magnetic separation, introducing a highly sensitive aptasensor, to analyze simultaneously AFB1 and OTA [23,83]. According to Guo et al. and Zhang et al., fluorescence aptasensors hold great potential for multi-mycotoxin determination [23,83].

#### 5.3.5. Surface Plasmon Resonance (SPR)

In this context, there is also an analytical strategy, Surface Plasmon Resonance (SPR). This technique has several advantages, such as high sensibility for multiple targets, good specificity, real-time monitoring, and high throughput detection [23,84]. Recently, another development of this method, an SPR-based aptasensor chip, has emerged in order to determine four mycotoxins, AFB1, OTA, ZEA, and DON [23,85]. At present, the majority of aspects, such as sensitivity, stability, time consumption, cost, and replicability, necessary for the detection of mycotoxins by aptamer biosensors are more successful than the ELISA, but it is essential to figure a method that could better compete with ELISA performance, namely regarding the simplification of the analytical principles and devices [23].

## 6. Multi-Mycotoxins Analytical Methodologies Applied to Groundnuts

A vast number of the studies mentioned in the literature determine a single mycotoxin. However, in the last few years, there has been a tendency to optimize and validate multi-mycotoxin analytical methods. Table 1 summarizes multi-analyte methods for mycotoxin determination in groundnuts (peanuts) and other types of food, such as nuts and dried fruits. According to the analytical methods summarized in Table 1, the most common analytical technique is liquid chromatography coupled with mass spectrometry. However, immunoassays such as ELISA and biosensors are also being developed and validated. The main differences among the selected methods for mycotoxin determination are efficiency, sensibility, selectivity, and the cost/efficiency relation, providing better detection and quantification of multi-mycotoxins. Concerning Table 1, among the extraction methods, SLE and QuEChERS are commonly used for multi-mycotoxin determination in groundnuts. These methods are also applied to nuts and dried foods (Table 1). Concerning chromatographic techniques, the majority of these methods are based in liquid chromatography coupled with mass spectrometry, allowing for the simultaneous analysis of different mycotoxins from different groups and using C18 analytical columns (Table 1).

Jinap et al. [89] obtained very low LODs (0.01–0.03 μg/kg) using an in-line immunoaffinity chromatographic method to determine aflatoxins in peanuts, dried figs, and paprika powder. Moreover, Wang et al. [95] obtained even lower LODs (0.00022–0.19 μg/kg) for AFB1, DON, T-2, and ZEA in peanuts and corn using a multiplex immunoassay–suspension array. Depending on the selected method, the specific mycotoxin, and the analyzed matrix, the LODs, LOQs, and precision can vary significantly (Table S1).

### 6.1. Mycotoxins in Groundnuts (Peanuts): RASFF Notifications

In order to provide valid information about contamination effects in the EU, the Rapid Alert System for Food and Feed (RASFF) provides food safety information through notifications for different countries from the EU or outside the EU.

Table S2 summarizes the RASFF notifications regarding mycotoxins in groundnuts. The period of the report is between 1 January 2024 and 31 December 2024, as obtained in RASFF [96], so it is possible to observe that all of the notifications are classified as serious risks, except for 8 from a total of 95, which are classified as potentially serious (Table S1). All the 2024 notifications regarding mycotoxins in peanuts were in food and just one was in feed (notification date: 21 November 24). Most of the notifications indicate that the contaminated groundnuts are from countries outside the EU, namely from Egypt and India. In 71% of cases, Netherlands was the notifying country, and in 22% of cases, it was Belgium. The highest value was found for groundnuts from Egypt, where an AFB1 level of 380 μg/kg was reported, as well as a total of 590 μg/kg for the sum of aflatoxins (B1, B2, G1, and G2). These aspects highlight the importance of developing and validating analytical methods to monitor mycotoxins in groundnuts.

### 6.2. Mycotoxins Detoxification

Normally, aflatoxins are resistant to common treatment strategies, for example pasteurization and sterilization. Their chemical structure makes them heat-resistant, meaning that conventional thermal treatments used in food processing (such as pasteurization at 72–85 °C or even sterilization at 121 °C for a short time) do not effectively degrade them. For this reason, it was necessary to develop effective physical, chemical, and biological methods to control the amount of mycotoxins in food [15,97,98,99,100,101]. It is recommended to focus on new technologies for the control of aflatoxins, which have potential field applications with the principal aim of protecting human and animal food/feed safety and health. Also, it is important to increase awareness about public health and prevention, raise economic benefits, and, at the same time, decrease costs [15].

Most of the techniques are applied to grain and cereal decontamination, but all the methods mentioned can be implemented for groundnuts (peanuts). The conventional decontamination techniques in cereals are mostly chemical, namely hydrogen peroxide, citric acid, lactic acid, propionic acid, and ozonated water [102]. According to some studies, there is a possibility to promote aflatoxin detoxification through the degradation of their structure using different gases or chemical agents that oxidize or hydrolase or through using thermal treatment. In relation to the hydrolysis, in acidic and alkaline conditions, this method of detoxification is able to open the lactone rings of aflatoxins to produce a water-soluble compound, the beta-keto acid, which is easily removed from the sample through rinsing with water. Concerning thermal inactivation, microwaving, extrusion and heating, irradiation of ultraviolet light, and absorption gases are the most prevailing physical techniques to detoxify aflatoxins. High temperatures (between 237 and 306 °C) or gamma radiation (called as cold process) will be used for decontamination to extend food shelf life by declining microbial density once this method is significantly effective in the degradation of mycotoxins [15]. There is also another type of method, bio-detoxification, which involves enzymes and microorganisms. In fermented food, lactic acid bacteria (LAB) and yeast strains can be used [15,103,104,105,106,107]. Additionally, ozone gas is a significant alternative for the detoxification of peanuts since it is efficient in preventing the growth of fungus that could produce aflatoxin and lower aflatoxin levels in peanut kernels [108].

Recent decontamination techniques have been developed without affecting the quality and nutrients, which can fulfill some objectives such as those pertaining to utility, environmentally friendliness, and cost-effectiveness. These decontamination techniques are UV radiation, gamma radiation, microwave UV radiation, extrusion, electron beam radiation, pulsed light, and heating [109]. In Table 2, it is possible to observe the effectiveness of each treatment through the percentage of mycotoxin reduction.

It is recommended to focus on new technologies for the effective control of aflatoxins, which have potential field applications with the principal aim of protecting human and animal food/feed safety and health. It is also important to increase awareness about public health and prevention, raising economic benefits and, at the same time, decreasing costs [15].

## 7. Conclusions and Future Perspectives

Peanuts (*Arachis hypogaea* L.) are commonly contaminated by mycotoxins, and, due to climate change, these contaminations could aggravate. The main extraction methods used for mycotoxins are SLE and QuEChERS, and the analytical techniques mainly used are LC coupled with mass spectrometry, immunoassays, namely ELISA, and biosensors for multi-mycotoxin determination.

According to the needs of multi-mycotoxin analysis, a possibility exists to use high-resolution mass spectrometry techniques, such as Orbitrap and time of flight (ToF); however, further studies are needed to assess their reliability, feasibility, and reproducibility in future quantifications. ELISA techniques are expected to continue to be used and developed.

Apta-based biosensors are promissory, neglecting higher sensibility. However, more research is needed to develop successful specific aptamers, aptasensors, and sensor arrays for multi-mycotoxin analyses.

For all these reasons presented, it is imperative to create and perform a technique capable of analyzing several mycotoxins simultaneously (ideally from different groups of mycotoxins) with high specificity, sensitivity, precision, and accuracy in different food matrices in order to be able to guarantee the safety of food samples, with reductions in time analysis, organic solvents (environmentally friendly), and costs.

## Figures and Tables

**Table 1:** Multi-mycotoxin determination in peanuts using different extraction methods and chromatographic conditions.

Type of Sample	Mycotoxins Analyzed	Clean-Up Methods	Extraction Methods	Detector	Conditions	Analytical Column	Internal Standard	LOD (μg/kg)	LOQ (μg/kg)	Ref.
Peanut Pistachio Wheat Maize Cornflakes Raisin Fig	AFB1, AFB2, AFG1, AFG2, OTA, DON, FB1, FB2, ZEA, H-2 Toxin, HT-2 Toxin,	-	Sample quantity: 25 g Extraction: 100 mL acetonitrile/water (80:20 *v*/*v*) on horizontal shaker for 2 h; 1 mL of the extract is diluted and mixed with 3 mL of water and filtered	LC-MS/MS	Mobile phase: solvent A H_2_O with 0.1% formic acid; solvent B acetonitrile with 0.1% formic acid Gradient program: 90% A at 0 min Flow rate: 0.3 mL/min Capillary voltage: 2.5 kv Desolvation temperature: 450 °C Desolvation gas flow: 600 L/h Collision gas pressure: 0.8 bar Ionization: ESI source in positive mode	Alltima C18 (150 × 3.2 mm, 5 μm)	-	0.5–75	1.0–150	[86]
Peanut Pistachio Almond	AFB1, AFB2, AFG1, AFG2, AFM1, OTA, FB1, FB2, ZEA, H-2 Toxin, HT-2 Toxin,	QuEChERS	Sample quantity: 5 g Extraction: 10 mL of Milli-Q water, 10 mL of ACN-formic acid (99.9/0.1 (*v*/*v*)), 4 g of anhydrous MgSO_4_, 1 g NaCl, 1 g of sodium citrate, 0.5 g of disodium hydrogen citrate sesquihydrate on centrifugation for 5 min; dSPE using EMR-Lipid activated with 5 mL of water shaken, 5 mL of organic extract centrifuged for 5 min; collected 5 mL of supernatant with 0.4 g NaCl, 1.6 g anhydrous MgSO_4_ centrifuged for 5 min; dilution 1:25 with water followed by filtration PTFE filters	HPLC- Orbitrap MS	Mobile phase: solvent A H_2_O with 0.1% formic acid (*v*/*v*) and solvent B MeCN with 0.1% formic acid (*v*/*v*) Gradient program: 0–5 min 4% B, 5–20 min 100% B, 20–24 min 100% B, 24–28 min 2% B for 10 min Flow rate: 200 nL/min Injection volume: 100 nL Capillary temperature: 250 °C Capillary voltage: 2.2 kV Column temperature: 25 °C Autosampler temperature: 7 °C Ionization: ESI source in positive mode	EASY-Spray PepMap C18 Nano column (75 μm × 150 mm, 3 μm)	-	-	0.05–5	[87]
Peanuts, Treenuts Cereals, Dried fruits, Spices	AFB1, AFB2, AFG1, AFG2	SPME	Sample quantity: 0.5 g Extraction: 1 mL of 80% methanol in water (80:20 *v*/*v*) centrifuged for 5 min, supernatant used directly in-tube SPME	HPLC-MS	Mobile phase: methanol/acetonitrile (69/40 *v*/*v*): 5 mM ammonium formate (45:55) Gradient program isocratic mode Flow rate: 1.0 mL/min Injection volume: 10 μL Column temperature: 40 °C Capillary voltage: 2500 V Drying gas flow: 13 L/min Drying gas temperature: 350 °C Gas pressure: 30 psi Ionization: ESI source in positive mode	Zorbax Eclipse XDB-C8 column 150 mm × 4.6 mm 5 μm	AFM1	0.04	0.05	[81]
Almonds, Hazelnuts, Peanuts, Pistachios, Walnuts	AFB1, AFB2, AFG1, AFG2, ZEA	QuEChERS	Sample quantity: 2 g Extraction: 10 mL of acetonitrile: water (80:20 *v*/*v*), 4 g Na_2_SO_4_ anhydrous salt, and 1 g NaCl centrifugation for 10 min; 3 mL of supernatant, 100 mg of C18, centrifugated for 10 min and filtered	UHPLC-MS/MS	Mobile phase: A: methanol; B: aqueous solution of ammonium formate 5 mM Gradient program Flow rate: 0.2 mL/min Injection volume: 5 μL Column temperature: 25 °C Capillary voltage: 3500 V Nozzle voltage: 500 V Sheath gas temperature: 400 °C Sheath gas flow: 11 L/min Gas temperature: 325 °C Gas flow: 5 L/min Gas pressure: 45 psi Ionization: ESI source in + mode for AFB1, AFB2, AFG1, and AFG2; ESI source in—mode for ZEA	Zorbax plus C18 column 100 × 2.1 mm, 1.8 μm	-	-	0.5–1.0	[88]
Apple juice, Grape juice, Orange juice, Pomegranate juice, Raisin, Dried-fig, Wheat flour, Barley flour, Peanuts, Pistachios, Chili, Mixed spice	AFB1, AFB2, AFG1, AFG2, OTA	QuEChERS	Sample quantity: 2.5 g Extraction: 10 mL acetonitrile, 10 mL water with 0.2% formic acid, rotation for 30 min; 4 g of magnesium sulfate, 1 g of sodium chloride, 1 g of sodium citrate, 0.5 g of sodium hydrogen citrate sesquihydrate, centrifugation; 2 extractions with 20 mL hexane; dSPE with supernatant, 150 mg C18, 900 mg magnesium sulfate, centrifugation, 2 washes with acetonitrile	LC-MS/MS	Mobile phase: water Flow rate: 0.2 mL/min Injection volume: 4 μL Column temperature: 30 °C Gas temperature: 250 °C Gas flow: 14 L/min Gas pressure: 25 psi Ionization: ESI source in positive mode	ODS CIS 150 mm × 2.1 mm, 5 μm	-	0.08–0.09	0.10–0.20	[24]
peanuts, dried figs, and paprika powder	AFB1, AFB2, AFG1, AFG2	in-line IAC	Sample quantity (peanut): 5 g Extraction: 1 g of NaCl, 30 mL of methanol/water (80:20), filtration; 10 mL of filtrate diluted with 60 mL of PBS Sample quantity (dried figs): 40 g Extraction: 5 g of NaCl, 200 mL of methanol/water (80:20) + 100 mL n- hexane, filtration; 10 mL of filtrate diluted with 60 mL of PBS	HPLC-FLD	Mobile phase: methanol/acetonitrile/water (30/20/50, *v*/*v*/*v*), 300 mL/L of nitric acid (HNO_3_), and 50 mg/L of KBr Binding buffer: 0.01 M Na_2_HPO_4_, 0.15 M NaCl (in water, pH adjusted to 7.0) Flow rate: 0.25 mL/min. Elution buffer contained acetonitrile/water (20/80, *v*/*v*) at a flow rate of 0.8 mL/min	reverse phase Genesis C18 HPLC column 25 cm × 4.6 mm, 4 mm	-	0.01–0.03	0.02–0.05	[89]
peanut, corn, wheat	AFB1, AFB2, AFG1, AFG2, OTA ZEA, T-2 toxin	mIAC.	Sample quantity: 20 g Extraction: ACN/water/acetic acid (80:19:1, *v*/*v*/*v*, 100 mL), supernatant filtrated and diluted with PBS at the ratio of 1:3	HPLC-MS/MS	Elution A: water + 0.05% formic acid Elution B: acetonitrile + 0.05% formic acid Flow rate: 250 μL/min 10 μL sample solution was injected into HPLC MS: triple quadrupole coupled + electrospray interface (ESI) Spray voltage: 4.0 kV for ESI+ and −3.0 kV for ESI− Capillary temperature: 350 °C Sheath gas pressure (N2): 30 units Auxiliary gas pressure (N2): 5 units Collision gas (Ar): 1.5 mTorr Scan time: 0.1 s	Thermo Scientific C18 column (Hypersil Gold, 100 mm × 2.1 mm, 3.0 μm) at 35 °C	-	0.04–0.4	0.3–0.12	[90]
spiked peanut and corn	AFB1 AFB2	multifunctional	Sample quantity: 25 g Extraction: 100 mL acetonitrile/deionized water (84:16), filtered, 9 mL of filtrate transferred into a test tube and pressed through Mycosep^®^ #226 AflaZon clean-up cartridge, 2 mL removed and evaporated to dryness. Reconstituted into 200 μL of methanol, vortexed and filtered through 0.45 μm nylon	HPLC-FLD	Mobile phase: deionized water/acetonitrile/methanol (60:20:20) Flow rate: 1.0 mL/min Injection volume: 20 μL Fluorescence detector: 375 nm (excitation), 440 nm (emission)	Platinum C18 column (250 × 4.6 mm id, 5 μm) at 40 °C	-	-	-	[91]
Rice, peanut, wheat, and maize	AFB1, AFB2, AFG1, AFG2, FB1, FB2, DON, ZON, OTA, T- 2 and HT-2	IAC	Sample quantity: 5 g Extraction: 25 mL PBS buffer, Extraction solution A: Filtered 17.5 mL of PBS-extracted supernatant Extraction solution B: 7.5 mL methanol added into the remaining PBS-extracted supernatant. 10 mL added to 90 mL PBS buffer to fix the volume at 100 mL	UHPLC-MS/MS	Injection volume: 10 μL Waters Zevo triple quadrupole tandem mass spectrometer To maximize analysis parameters, 1.0 μg/mL of each toxin standard sample at 10 μL/min was injected Capillary voltage: 2.5 kV Extractor voltage: 2.5 V Source temperature: 150 °C Desolvation temperature: 500 °C Desolvation gas (nitrogen) flow: 1000 L/h	ACQUITY BEH C18 (2.1 × 100 mm, 1.7 μm particle size)—30 °C	-	0.05–1.0	0.5–20	[92]
Peanuts	AFB1, AFB2, AFG1, AFG2, ZEN, OTA	-	Sample quantity (Milled peanut samples): 10.0 g Extraction: 50.0 mL methanol/water (80:20, *v*/*v*), 5.00 mL extracting solution was moved into a 10 mL glass and centrifuged, followed by DLLME	HPLC-FLD	Mobile phase: A—acetic acid/water (1:99, *v*/*v*), B- Acetonitrile Flow rate: 1.00 mL/min Injection volume: 10.0 μL	HLPC column Inertsil^®^ DS-3 (4.6 × 250 mm, 5 μm)	-	0.03–1.0	0.5–2.0	[93]
maize, peanut, rice orange juice, grape juice	AFB1, OTA, FB1	-	Samples quantity: 6 g of the mashed blank crops (maize, peanut, and rice) Extraction; 30 mL of methanol/water (7:3, *v*/*v*). All extract samples were pretreated by simple filter processing	MagQBD-ICA	Components: nitrocellulose (NC) membrane containing three test lines (T lines) and a control line (C line), sample pad (MagQBD immuno-complex loading), and absorbent pad AFB1-BSA (0.8 mg/mL), OTA-BSA (0.6 mg/mL), and FB1-BSA (0.06 mg/mL) dissolved in PBS buffer (10 mM, pH 7.4), sprayed onto the NC membrane Goat anti-mouse IgG antibody (0.4 mg/mL) applied to the NC membrane (C line) The NC membrane was placed in a drying oven at 37 °C for 3 h, sample pad + absorbent pad assembled together on the plastic backing card, cut (3 mm strips), and stored in a vacuum desiccator until use	--	-	0.05–0.5	-	[94]
peanuts, corn	AFB1, DON, T-2, ZEA	-	Samples quantity: 2 g Extraction; 10 mL methanol: water (20:80, *v*/*v*- corn powder, 40:60, *v*/*v*- peanut extract) The mixture was blended. Supernatant was collected. The extract was diluted 10 times (Ab dilution buffer), stored at 4 °C.	multiplex immunoassay-suspension array	All mycotoxin antigens were successfully bound to the microsphere using 1-ethyl-3-(3-dimethylaminopropyl) carbodiimide hydrochloride Chessboard trituration was used to determine the best antibody concentrations and biotin—rabbit anti-goat igG Using indirect competitive immunoassay, the four mycotoxins were quantitatively detected	-	-	0.00022–0.19	-	[95]

**Table 2:** Influence of different decontamination techniques.

Mycotoxin	Sample	Decontamination Technique	% Reduction in Mycotoxins	Reference
AFB1	Peanut surface	UV radiation	100	[110]
	Corn and walnut	Gamma radiation	>80	[111]
	Peanuts	Microwave heating (360, 480 and 600 W)	59–67	[112]
		Gamma radiation	20–43	
		Ozonation	30–25	[108]
	Peanut meal	Extrusion	77	[113]
	Brazil nut	Gamma radiation	71	[114]
		Electron bean radiation	84	
	Pistachio nuts (mixed with lemon juice and citric acid)	Heating	93	[115]

## Data Availability

No new data were created or analyzed in this study. Data sharing is not applicable to this article.

## Supplementary Materials

The following supporting information can be downloaded at: https://www.mdpi.com/article/10.3390/foods14050902/s1, Table S1. Key analytical parameters for the determination of multi-mycotoxins in peanuts and peanut-based samples [83,116,117,118,119,120,121,122,123,124,125,126,127,128,129,130,131,132,133,134]; Table S2. Notifications of mycotoxins in groundnuts according to RASFF in 2024.
