# Supplementary material for: Peanuts (Arachis hypogaea L.) and Mycotoxins: Analytical Approaches, Prevalence, and Innovative Detoxification
Source: Foods. 2025 Mar 6;14(5):902. doi: 10.3390/foods14050902 (PMC11898414; doi:10.3390/foods14050902)
Supplement: Supplementary file 1 [file foods-14-00902-s001.zip › foods-3429600-supplementary.pdf]

Supplementary Material

Table S1. Key analytical parameters for the determination of multi-mycotoxins in peanuts and peanut-based samples.

Ref.	Year	Analitical Tecniques	Sample(s) (origin country)	Target Analyte	LOD µg/kg)	LOQ (µg/kg)	Recovery (%)	RSD (%) Intra-day (inter-day)
(116)	2013	**UHPLC-MS/MS**	Peanut	AFB1	0.05	0.1	**71**-74	4-7
				AFB2,	0.05	0.1	79-82	3-6
				AFG1	0.05	0.1	73-78	4-8
				AFG2	0.05	0.1	72-91	6-8
				FB1	5	10	75	10
				FB2	5	10	72	9
				FB3	5	10	78	10
				DON	1	5	74-83	5-7
				ZON	1	5	75-80	4-6
				HT-2	0.5	1	91-93	3
				T-2	0.5	1	88-95	4-5
				OTA	0.1	0.25	77-**103**	3-6
(117)	2013	**UHPLC-MS/MS**	Peanut (Turkey) (n=15)	191 fungal metabolites	Due to the numerous number of target analytes this information is not displayed in this table			
(118)	2013	**UHPLC-MS/MS**	Peanut (Spain)	AFB1,	0.21,	0.71	71.8-75.4	5.1-5.9
				AFB2	0.29	0.97	80.3-91.6	4.6-.6.6
				AFG1	0.18	0.61	71.4-74.4	3.0-7.2
				AFG2	0.29	0.95	84.3-94.0	6.3-8.8
				FB1,	0.47	1.57	66.9-77.1	4.0-8.3
				FB2,	0.19	0.65	78.1-91.2	2.7-7.7
				DON	9.68	32.6	82.8-93.0	3.7-6.9
				HT-2	1.13	3.77	79.0-83.4	2.9-7.8
				T-2	1.42	4.74	93.1-**96.1**	3.5-7.2
				OTA	0.17	0.57	83.6-89.0	1.4-8.6
				F-X	45.1	150	73.0-89..3	4.2-5.3

				STE	0.18	0.61	70.0-85.1	5.9-7.4
				ZEN	6.05	20.2	81.2-94.2	0.6-8.5
				CIT	0.52	1.71	**69.5**-80.1	2.9-8.9
(Liao et al., 2013)	2013	**LC-MS/MS**	Peanut flour,peanut butter, and raw peanut	26 mycotoxins	Due to the numerous amounts of target analytes, this information is not displayed in this table			
(93)	2013	**UHPLC-MS/MS**	Penuts (China)	AFB1	0.05	0.5-20	84.5	8.2 (18.5)
				AFB2	0.05		89.1	14.1 (19.4)
				AFG1	0.05		92.4	13.0 (8.8)
				AFG2	0.05		94.0	12.9 (7.0)
				FB1	1		98.4	14.4 (4.3)
				FB2	0.5		77.1	9.4 (5.4)
				DON	0.5		87.2	6.7 (3.8)
				ZON	1		**14.3**	38.4 (36.5)
				OTA	0.5		73.4	5.8 (14.9)
				T-2	0.1		**104.3**	8.2 (19.3)
				HT-2	0.5		102.0	18.1 (6.7)
(119)	2013	**Chip-nanoLC/QqQ-MS**	Peanut, peanut powder, peanut butter,	AFB1	0.004	0.048	92.9-98.7	4.4–4.0 (2.9–5.4)
				AFB2	0.006		93.4-96.4	2.3–9.5 (2.8–6.5)
				AFG1	0.007		90.9-92.0	3.1–4.8 (2.3–6.3)
				AFG2	0.007		91.5-94	4.1–6.3 (3.8–5.8)
				AFM1	0.008		**90.8-100.4**	3.9–8.0 (2.7–6.6)
(120)	2013	**Multiplex competition immunoassay system**	Peanut	AFB1	0.5	–	80.3-116.7	–
				CIT	1		**80.1**-105.5	
				FB1	0.8		87.4-**117.2**	
(121)	2013	**LC-ESI-QTOF-MS/MS**	**Peanut,** peanut butter (Malasya)	AFB1	0.117	0.391	76.1-87.0	8.5-9.6
				AFB2	0.141	0.469	69.0-103.7	7.3-13.1
				AFG1	0.176	0.586	72.8-84.6	10.5-13.2
				AFG2	0.211	0.703	**67.0-103.9**	8.3-12.6
(122)	2013		Peanut	AFB1	0.52-0.36	–	77-85	3.82-7.94

		**icELISA based on MAb**		AFM1			–	–
(123)	2015	**UHPLC-MS/MS**	Roasted and raw peanut	AFB1	0.13	0.19	83.5-89.8	5.4
				AFB2	0.21	0.48	71.3-86.8	11.9
				AFG1	0.03	0.88	79.3-98.5	6.0
				AFG2	0.26	0.10	85.6-98.9	15.1
				AFM1	0.14	0.69	82.6-100.3	7.3
				AFM2	0.06	0.43	83.5-89.8	4.8
(124)	2015	**TFC–LC–MS/MS**	Peanut butter	AFB1	1	0.5-4.0	96-100.5	1.9-6.5
				AFB2	1		94.0-102.2	2.3-8.5
				AFG1	1		92.4-96.8	1.6-3.1
				AFG2	2		89.7-94.2	3.7-5.4
(125)	2015	**UHPLC/Q-orbital trap MS**	Peanut	**26 mycotoxins**	Due to the numerous amounts of target analytes this information is not displayed in this table			
(82)	2016	**mIAC-HPLC–MS/MS**	Peanut (China)	AFB1	0.1	0.3	98.6	2.0–3.2 (6.1–8.5)
				AFB2	0.04	0.12	100.6	
				AFG1	0.1	0.3	98.2	
				AFG2	0.04	0.12	101.7	
				OTA	0.2	0.6	97.5	
				ZEN	0.1	0.3	97.7	
				T-2	0.4	0.12	98.2	
(97)	2017	**HPLC-FLD**	Peanut (China)	AFB1	0.10	0.5	83.2-96.2	4.0-6.8
				AFB2	0.03	0.15	83.1-98.5	4.5-7.3
				AFG1	0.10	0.5	89.8-96.8	4.4-6.9
				AFG2	0.03	0.15	89.7-99.3	3.8-7.1
				OTA	0.3	0.5	87.3-98.4	3.4-4.8
				ZEN	1.0	2.0	87.3-99.5	2.8-4.5
(126)	2017	**QDNBs based ICA strip**	Peanut	AFTs	2.2	–	91.3-106.3	4.6-11.8

(127)	2017	**ToxiMet system**	Peanut paste (Ivory Coast)	AFB1	0.20	0.67	–	–
				AFB2	0.12	0.40		
				AFG1	0.21	0.72		
				AFG2	0.04	0.14		
(33)	2018	**UHPLC-MS/MS**	Peanut paste (Ivory Coast)	**77 mycotoxins**	Due to the numerous amount of target analytes, this information is not displayed in this table			
(128)	2018	**LC-MS/MS**	Peanuts (Portugal)	DON	1.25	1	80-94	5-12 (8-10)
				FUS X	5	3	95-115	5-22 (8-18)
				NEO	1.25	0.5	71-93	4-9 (9-12)
				3Ac-DON	5	1.5	67-89	2-11 (8-15)
				15Ac-DON	5	2	63-86	4-10 (6-12)
				AfG2	1.25	0.4	62-91	11-16 (15-19)
				AfG1	1.25	0.4	63-91	6-18 (9-15)
				AFB2	1.25	0.5	67-89	8-13 (9-12)
				AfB1	1.25	0.5	56-84	13-18 (11-18)
				DAS	1.25	0.3	74-95	5-20 (9-17)
				FB1	5	2	59-78	8-10 (10-14)
				T-2	1.25	1.5	65-96	6-16 (4-12)
				HT-2	1.25	1	73-90	5-17 (6-15)
				OTA	5	2	57-102	7-16 (10-12)
				FB2	5	3	65-89	9-18 –(11-13)
				ZEA	5	3.5	72-92	4-14 (9-10)
(88)	2019	**UHPLC-QqQ-MS/M**	Peanuts (Spain)	AFB1	–	0.5	89.1-100.6	6.7 (8.9)
				AFB2		0.5	88.3-99.7	3.8 (11.1)
				AFG1		0.5	90.1-100.3	3.8 (14.5)
				AFG2		0.5	92.7-103.0	10.8 (15.4)
				α-ZOL		1	73.6-116.3	3.5 (18.5)
				ZEA		1	87.6-99.9	4.6 (17.0)

(86)	2019	**nanoflow LC-HRM**	Peanut (Spain)	3-ADON	–	0.543	–	–
				AFB1		0.040		
				AFB2		0.041		
				AFG1		0.003		
				AFG2		0.005		
				AFM1		0.004		
				α-ZOL		0.021		
				DAS		0.072		
				ERGC		0.074		
				FB1		0.032		
				FB2		0.331		
				GLI		0.340		
				HT-2		0.385		
				OTA		0.014		
				T—2		0.505		
				ZEN		0.010		
(129)	2019	**LC-electrospray ionization-MS/MS**	Peanut oil	AFB1	0.03	0.1	107-113	<20
				AFB2	0.03	0.1	106-110	
				AFG1	0.03	0.1	104-110	
				AFG2	0.06	0.2	106-110	
				DON	1	3.3	106-108	
				FB1	1.3	4.3	107-109	
				FB2	0.9	2.9	110-119	
				FB3	0.8	2.6	115-142	
				OTA	1.5	4.8	90-92	
				HT-2	0.7	2.2	101-110	
				T-2	0.7	2.4	97-105	
				ZON	0.9	6.4	106-120	
(130)	2020	**Electrochemical biosensor based on *E. coli***	Peanut oil	ABF1	1	–	90-112	0.34-8.99
				ZEN	6		92-94.3	3.44-11.93

(131)	2020	**LC–MS/MS QTRAP system in multiple reaction monitoring**	Peanut kernel, peanut butter, and roasted peanuts (China)	AFB1	0.20	0.60	85.1-99.6	4.4-5.9 (4.4-5.5)
				AFB2	0.20	0.60	82.6-99.1	1.6-7.6 (5.5-8.5)
				AFG1	0.26	0.78	105.3-113.6	3.6-4.7 (5.6-7.4)
				AFG2	0.26	0.78	81.9-93.4	3.9-7.6 (3.9-6.2)
(132)	2020	**Lateral Flow Strip Based on a Truncated Aptamer-Complementary Strand**	Peanut (China) (n=9)	AFB1	0.16	0.5	96.5-103.7	2.9-4.1
				AFB2			1.01.0-108.7	0.8-1.4
(95)	2022	**MagQBD- ICA**	Peanut	AFB1	0.00042	–	88.85-108.12	1.93-7.92
				OTA	0.011		92.60-97.22	6.07-9.54
				FB1	0.0042		95.98-108.69	7.34-9.68
(133)	2022	**IMSPE-UHPLC-MS/MS**	Peanut (China)	AFB1	0.01	0.04	85.2-100.3	2.9-4.8 (3.0-6.1)
				AFB2	0.01	0.04	84.5-97.6	3.5-9.7 (5.2-9.5)
				AFG1	0.01	0.04	86.4-112.7	2.9-6.3 (3.5-7.0)
				AFG2	0.01	0.04	93.5-102.6	2.8-4.9(2.7-15.2)
				FB1	0.02	0.06	88.3-97.3	2.8-6.11 (3.2-8.5)
				T-2	0.05	0.16	98.1-107.2	1.6-5.3 (4.7-7.2)
				CPA	0.02	0.06	89.3-94.8	3.9-6.9 (4.1-14.2)
(134)	2022		Peanut	AFB1	0.03	0.09	99.44-100.05	1.99-7.93 (0.29-5.28)

		**Ultra-fast RP-HPLC-FD-DAD**	(Nigeria) (n=30)	AFB2	0.02	0.07	100.16-100.82	2.84-14.06 (1.32-4.91)
				AFG1	0.04	0.10	99.57-100.08	2.50-10.88 (0.38-4.95)
				AFG2	0.03	0.08	100.02-100.39	2.98-7.99(1.12-10.44)

References (Supplementary material- Table S1)

116. Vaclavikova, M., Macmahon, S., Zhang, K., & Begley, T. H. Application of single immunoaffinity clean-up for simultaneous determination of regulated mycotoxins in cereals and nuts. *Talanta*, 2013, *117*, 345–351. https://doi.org/10.1016/J.TALANTA.2013.09.007

117. Varga, E., Glauner, T., Berthiller, F., Krska, R., Schuhmacher, R., & Sulyok, M. Development and validation of a (semi-)quantitative UHPLC-MS/MS method for the determination of 191 mycotoxins and other fungal metabolites in almonds, hazelnuts, peanuts and pistachios. *Analytical and Bioanalytical Chemistry*, 2013, *405*(15), 5087–5104. https://doi.org/10.1007/S00216-013-6831-3/TABLES/7

118. Arroyo-Manzanares, N., Huertas-Pérez, J. F., Gámiz-Gracia, L., & García-Campaña, A. M. A new approach in sample treatment combined with UHPLC-MS/MS for the determination of multiclass mycotoxins in edible nuts and seeds. *Talanta*, 2013, *115*, 61–67. https://doi.org/10.1016/J.TALANTA.2013.04.024

119. Liu, H. Y., Lin, S. L., Chan, S. A., Lin, T. Y., & Fuh, M. R. Microfluidic chip-based nano-liquid chromatography tandem mass spectrometry for quantification of aflatoxins in peanut products. *Talanta*, 2013, *113*, 76–81. https://doi.org/10.1016/J.TALANTA.2013.03.053

120. Deng, G., Xu, K., Sun, Y., Chen, Y., Zheng, T., & Li, J. High Sensitive Immunoassay for multiplex mycotoxin detection with photonic crystal microsphere suspension array. *Analytical Chemistry*, 2013, *85*(5), 2833–2840. https://doi.org/10.1021/ac3033728

121. Sirhan, A. Y., Tan, G. H., & Wong, R. C. S. Determination of aflatoxins in food using liquid chromatography coupled with electrospray ionization quadrupole time of flight mass spectrometry (LC-ESI-QTOF-MS/MS). *Food Control*, 2013, *31*(1), 35–44. https://doi.org/10.1016/J.FOODCONT.2012.09.016

122. Jiang, W., Wang, Z., Nölke, G., Zhang, J., Niu, L., & Shen, J. Simultaneous Determination of Aflatoxin B1 and Aflatoxin M1 in Food Matrices by Enzyme-Linked Immunosorbent Assay. *Food Analytical Methods*, 2013, *6*(3), 767–774. https://doi.org/10.1007/S12161-012-9484-5/FIGURES/4

123. Sartori, A. V., Swensson De Mattos, J., Souza, Y. P., Pereira, R., Santos, D., Heloísa, M., de Moraes, P., & Wanderley Da Nóbrega, A. Determination of aflatoxins M1, M2, B1, B2, G1 and G2 in peanut by modified QuEChERS method and ultra-high performance liquid chromatography-tandem mass spectrometry. *Vigilância Sanitária Em Debate: Sociedade, Ciência & Tecnologia*, 2015, *3*(3), 115–121. https://doi.org/10.3395/2317-269x.00406

124. Fan, S., Li, Q., Zhang, X., Cui, X., Zhang, D., & Zhang, Y. Simultaneous determination of aflatoxin B1, B2, G1, and G2 in corn powder, edible oil, peanut butter, and soy sauce by liquid chromatography with tandem mass spectrometry utilizing turbulent flow chromatography. *Journal of Separation Science*, 2015, *38*(8), 1310–1317. https://doi.org/10.1002/JSSC.201401376

125. Liao, C. D., Wong, J. W., Zhang, K., Yang, P., Wittenberg, J. B., Trucksess, M. W., Hayward, D. G., Lee, N. S., & Chang, J. S. Multi-mycotoxin Analysis of Finished Grain and Nut Products Using Ultrahigh-Performance Liquid Chromatography and Positive Electrospray Ionization-Quadrupole Orbital Ion Trap High-Resolution Mass Spectrometry. *Journal of Agricultural and Food Chemistry*, 2015, *63*(37), 8314–8332. https://doi.org/10.1021/JF505049A/SUPPL_FILE/JF505049A_SI_002.PDF

126. Ouyang, S., Zhang, Z., He, T., Li, P., Zhang, Q., Chen, X., Wang, D., Li, H., Tang, X., & Zhang, W. An On-Site, Ultra-Sensitive, Quantitative Sensing Method for the Determination of Total Aflatoxin in Peanut and Rice Based on Quantum Dot Nanobeads Strip. *Toxins*, 2017, *9*(4), 137. https://doi.org/10.3390/TOXINS9040137

127. Campbell, K., Ferreira Cavalcante, A. L., Galvin-King, P., Oplatowska-Stachowiak, M., Brabet, C., Metayer, I., Montet, D., Haughey, S. A., & Elliott, C. T. Evaluation of an alternative spectroscopic approach for aflatoxin analysis: Comparative analysis of food and feed samples with UPLC–MS/MS. *Sensors and Actuators, B: Chemical*, 2017, *239*, 1087–1097. https://doi.org/10.1016/J.SNB.2016.08.115
128. Cunha, S. C., Sá, S. V. M., & Fernandes, J. O. Multiple mycotoxin analysis in nut products: Occurrence and risk characterization. *Food and Chemical Toxicology*, 2018, *114*, 260–269. https://doi.org/10.1016/J.FCT.2018.02.039
129. Zhang, K., & Xu, D. Application of Stable Isotope Dilution and Liquid Chromatography Tandem Mass Spectrometry for Multi-Mycotoxin Analysis in Edible Oils. *Journal of AOAC INTERNATIONAL*, 2019, *102*(6), 1651–1656. https://doi.org/10.1093/JAOAC/102.6.1651
130. Chen, Y., Yang, Y., Wang, Y., Peng, Y., Nie, J., Gao, G., & Zhi, J. Development of an Escherichia coli-based electrochemical biosensor for mycotoxin toxicity detection. *Bioelectrochemistry*, 2020, *133*. https://doi.org/10.1016/J.BIOELECHEM.2019.107453
131. Lv, S., Wang, H., Yan, Y., Ge, M., & Guan, J. Quantification and confirmation of four aflatoxins using a LC–MS/MS QTRAP system in multiple reaction monitoring, enhanced product ion scan, and MS3 modes. *European Journal of Mass Spectrometry*, 2020, *26*(1), 63–77. https://doi.org/10.1177/1469066719866050
132. Zhao, Z., Wang, H., Zhai, W., Feng, X., Fan, X., Chen, A., & Wang, M. A Lateral Flow Strip Based on a Truncated Aptamer-Complementary Strand for Detection of Type-B Aflatoxins in Nuts and Dried Figs. *Toxins*, 2020, *12*(2), 136. https://doi.org/10.3390/TOXINS12020136
133. Wang, W., Zhang, Q., Ma, F., & Li, P. Simultaneous determination of aflatoxins, fumonisin B1, T-2 and cyclopiazonic acid in agri-products by immunomagnetic solid-phase extraction coupled with UHPLC-MS/MS. *Food Chemistry*, 2022, *378*. https://doi.org/10.1016/J.FOODCHEM.2021.132020
134. Salisu, B., Anua, S. M., Wan Rosli, W. I., Mazlan, N., & Haron, R. Ultra-fast RP-HPLC-FD-DAD for quantification of total aflatoxins in maize, rice, wheat, peanut and poultry feed without sample clean up, and population exposure risk assessment in Katsina, Nigeria: an optimization study. *Journal of Environmental Science and Health, Part B*, 2022, *57*(7), 541–553. https://doi.org/10.1080/03601234.2022.2073151

Table S2. Notifications of mycotoxins in groundnuts according to RASFF in 2024.

Subject	Date	Origin	Notifying country	Classification	Decision	Level (µg/kg)
Aflatoxin in groundnuts from Egypt	20-dec-2024	Czech Republic	Netherlands	alert notification	serious	AFB1: 12; Sum AFs: 14
Aflatoxin in groundnuts from Argentina	16-dec-2024	Argentina	Netherlands	information notification for attention	serious	AFB1: 6.7; Sum AFs: 7.7
Aflatoxin in USA groundnuts	13-dec-2024	United States	Netherlands	border rejection notification	serious	AFB1: 20; Sum AFs: 22
Aflatoxin in Indian groundnuts	29-nov-24	India	Netherlands	border rejection notification	serious	AFB1: 8.4; Sum AFs: 8.4
Groundnuts China Aflatoxin	29-nov-24	China	Netherlands	border rejection notification	serious	AFB1: 12; Sum AFs: 13
Aflatoxin in groundnuts from Egypt via Germany	28-nov-24	Egypt	Netherlands	alert notification	serious	AFB1: 2.4-6.3; Sum AFs: 8,7
Aflatoxins in blanched organic groundnuts, from Egypt	27-nov-24	Egypt	Netherlands	alert notification	serious	AFB1: 10.6; Sum AFs: 15.2
Aflatoxin in groundnuts from Brazil	27-nov-24	Brazil	Netherlands	information notification for attention	serious	AFB1: 4.5; Sum AFs: 5.3
Aflatoxins in variety of blanched organic groundnuts, from Egypt	26-nov-24	Egypt	Netherlands	alert notification	serious	AFB1: 4.6-6.2; Sum AFs: 5.2-7.1
**Groundnuts USA Feed Aflatoxin***	**21-nov-24**	**United States**	**Netherlands**	**border rejection notification**	**potentially serious**	**AFB1: 150**
Aflatoxin in groundnuts from Argentina	12-nov-24	Argentina	Netherlands	information notification for attention	serious	AFB1: 3.9; Sum AFs: 4.5
Aflatoxin in groundnuts, from Argentina	6-nov-24	Argentina	Netherlands	information notification for attention	serious	AFB1: 15.21; Sum AFs: 17.5
Groundnuts USA Aflatoxin	5-nov-24	United States	Netherlands	border rejection notification	potentially serious	AFB1: 4.6
Aflatoxin in unprocessed groundnuts, from Argentina	5-nov-24	Argentina	Netherlands	information notification for attention	serious	AFB1: 4.7; Sum AFs: 4.7

Aflatoxin B1 15.7 ppb and Aflatoxin total 18.0 ppb in organic blanched groundnuts	4-nov-24	Egypt	Netherlands	alert notification	serious	AFB1: 15.7; Sum AFs: 18
Aflatoxins in organic groundnuts from Egypt.	31-oct-2024	Egypt	Belgium	border rejection notification	serious	AFB1: 22-40; Sum AFs: 24-43
Aflatoxins in organic groundnuts from Egypt.	31-oct-2025	Egypt	Belgium	border rejection notification	serious	AFB1: 27; Sum AFs: 30
Aflatoxins in organic groundnuts from Egypt.	31-oct-2026	Egypt	Belgium	border rejection notification	serious	AFB1: 87-96; Sum AFs: 97-100
Aflatoxins in organic groundnuts from Egypt.	31-oct-2027	Egypt	Belgium	border rejection notification	serious	AFB1: 13-160; Sum AFs: 14-170
Aflatoxins in organic groundnuts from Egypt.	31-oct-2028	Egypt	Belgium	border rejection notification	serious	AFB1: 21-33; Sum AFs: 22-35
Aflatoxins in organic groundnuts from Egypt.	31-oct-2029	Egypt	Belgium	border rejection notification	serious	AFB1: 70-100; Sum AFs: 77-110
Aflatoxins in groundnuts from Egypt.	31-oct-2030	Egypt	Belgium	border rejection notification	serious	AFB1: 110-150; Sum AFs: 120-160
Aflatoxins in groundnuts from Egypt.	31-oct-2031	Egypt	Belgium	border rejection notification	serious	AFB1: 58-82; Sum AFs: 65-92
Aflatoxins in organic groundnuts from Egypt.	31-oct-2032	Egypt	Belgium	border rejection notification	serious	AFB1: 37-42; Sum AFs: 40-46
Aflatoxins in organic groundnuts from Egypt.	31-oct-2033	Egypt	Belgium	border rejection notification	serious	AFB1: 4.5
Aflatoxins in organic groundnuts from Egypt.	31-oct-2034	Egypt	Belgium	border rejection notification	serious	AFB1: 3.3
Aflatoxin in USA groundnuts	28-oct-2024	United States	Netherlands	border rejection notification	serious	AFB1: 54; Sum AFs: 64

Aflatoxin in groundnuts from USA	25-oct-2024	United States	Bulgaria	border rejection notification	serious	AFB1: 51.3; Sum AFs: 56.5
Aflatoxin in USA groundnuts	21-oct-2024	United States	Netherlands	border rejection notification	serious	AFB1: 24; Sum AFs: 26
Aflatoxin B1 and total in Groundnuts from Argentina	4-oct-2024	Argentina	Netherlands	information notification for attention	serious	AFB1: 7.8-14; Sum AFs: 8.7- 16
Aflatoxin B1 in groundnuts from Argentina	1-oct-2024	Argentina	Netherlands	information notification for attention	serious	AFB1: 4.8; Sum AFs: 13.7
Aflatoxin in USA groundnuts	27-sep-2024	United States	Netherlands	border rejection notification	serious	AFB1: 22; Sum AFs: 25
Aflatoxin in groundnuts from Nicaragua	20-sep-2024	Nicaragua	Netherlands	information notification for attention	serious	AFB1: 14; Sum AFs: 15
Aflatoxin in groundnuts from the United States	2-sep-2024	United States	Netherlands	alert notification	serious	AFB1: 7.3; Sum AFs: 8
Aflatoxin B1 in groundnuts from USA	26- aug-2024	United States	Netherlands	information notification for attention	serious	AFB1: 7.2; Sum AFs: 8.4
Aflatoxin in USA groundnuts	23- aug-2024	United States	Netherlands	border rejection notification	serious	AFB1: 4.1
Aflatoxine B1 in groundnuts from Argentina	16- aug-2024	Argentina	Netherlands	information notification for attention	serious	AFB1: 9.7; Sum AFs: 10.0
Aflatoxine B1 in groundnuts from the USA	16- aug-2024	United States	Netherlands	information notification for attention	serious	AFB1: 12.4; Sum AFs: 15.2
Aflatoxin B1 and total in Groundnuts from Argentina	12-aug-2024	Argentina	Netherlands	information notification for attention	serious	AFB1: 2.7; Sum AFs: 6.7
Aflatoxin in groundnuts	5-aug-2024	Argentina	Netherlands	information notification for attention	serious	AFB1: 4.5; Sum AFs: 8.3
Aflatoxin in USA groundnuts	19-jul-24	United States	Netherlands	border rejection notification	serious	AFB1: 4.3
Aflatoxin B1 and total aflatoxins in grounded red groundnuts from Uganda	16-jul-24	Uganda	Finland	border rejection notification	serious	AFB1: 115.8; Sum AFs: 248.6

Aflatoxin B1 and total in Groundnuts from Argentina	15-jul-24	Argentina	Netherlands	information notification for attention	serious	AFB1: 13; Sum AFs: 36
Aflatoxin B1 and total in Groundnuts from Argentina	15-jul-24	Argentina	Netherlands	information notification for attention	serious	AFB1: 110; Sum AFs: 130
Groundnuts USA Aflatoxin	3-jul-24	United States	Netherlands	border rejection notification	potentially serious	AFB1: 5.1
Aflatoxins in groundnuts in shell from Egypt	3-jul-24	Egypt	Germany	border rejection notification	serious	AFB1: 8.2; Sum AFs: 9.3
Aflatoxin in USA groundnuts	26-jun-24	United States	Netherlands	border rejection notification	serious	AFB1: 14; Sum AFs: 16
Aflatoxin in USA groundnuts	26-jun-24	United States	Netherlands	border rejection notification	serious	AFB1: 6.7; Sum AFs: 8.3
Groundnuts USA Aflatoxin	26-jun-24	United States	Netherlands	border rejection notification	serious	AFB1: 42; SUM AFS: 47
Groundnuts India Aflatoxin	12-jun-24	India	Netherlands	border rejection notification	serious	AFB1: 11; sum AFs: 12
Aflatoxins in organic groundnuts from Egypt.	12-jun-24	Egypt	Belgium	border rejection notification	serious	AFB1: 6.1 and 11; sum AFs: 6.1 and 12
Aflatoxins in organic groundnuts from Egypt.	12-jun-24	Egypt	Belgium	border rejection notification	serious	AFB1: 6.3 and 15; sum AFs: 7.4 and 18
Aflatoxins in organic groundnuts from Egypt.	12-jun-24	Egypt	Belgium	border rejection notification	serious	AFB1: 5.8; sum AFs: 5.9
Aflatoxin B1 in raw groundnuts from the USA	12-jun-24	United States	Netherlands	information notification for attention	serious	AFB1: 5.8; sum AFs: 5.9
Groundnuts USA aflatoxin	5-jun-24	United States	Netherlands	border rejection notification	potentially serious	AFB1: 78; sum AFs: 92
Aflatoxin in Indian groundnuts	4-jun-24	India	Netherlands	border rejection notification	serious	AFB1:56

Aflatoxins in shelled groundnuts from the United States	4-jun-24	United States	Netherlands	border rejection notification	serious	AFB1: 65; sum AFs: 74
Aflatoxin in Indian groundnuts	29-may-2024	India	Netherlands	border rejection notification	serious	AFB1: 17; sum AFs: 20
Aflatoxin in groundnuts	27- may-2024	United States	Netherlands	information notification for attention	serious	AFB1: 8.9; sum AFs: 9.5
Groundnuts USA Aflatoxin	27- may-2025	United States	Netherlands	border rejection notification	potentially serious	AFB1: 12; sum AFs: 14
Aflatoxins in groundnuts from the USA	27- may-2026	United States	Netherlands	border rejection notification	serious	AFB1: 5.2
Aflatoxin in USA groundnuts	23-may-2024	United States	Netherlands	border rejection notification	serious	AFB1: 4.7; sum AFs: 12
Aflatoxins in groundnuts from Egypt	23-may-2025	Egypt	Belgium	border rejection notification	serious	AFB1: 5.7
Groundnuts USA Aflatoxin	14-may-2024	United States	Netherlands	border rejection notification	serious	AFB1: 15; sum AFs: 18
Aflatoxin B1 and total aflatoxins in groundnuts from the United States	23-apr-2024	United States	France	border rejection notification	serious	AFB1: 19; sum AFs: 22
Aflatoxin B1 in groundnuts from Paraguay	22-apr-2024	Paraguay	Netherlands	information notification for attention	serious	AFB1:22
Aflatoxin in Egyptian groundnuts	11-apr-2024	Egypt	Netherlands	border rejection notification	serious	AFB1: 11; sum AFs: 11
Aflatoxin contamination in groundnuts	8-apr-2024	China	France	alert notification	serious	AFB1: 4.8 and 16.4; sum AFs: 20.8
Aflatoxin B1 in groundnuts from Paraguay	8-apr-2025	Paraguay	Netherlands	information notification for attention	serious	AFB1: 4.3; sum AFs: 4.6
Aflatoxin in USA groundnuts	29-mar-24	United States	Netherlands	border rejection notification	serious	AFB1: 21; sum AFs: 24
Aflatoxin B1 in groundnuts from Paraguay	28-mar-24	Paraguay	Netherlands	information notification for attention	serious	AFB1: 11.9; sum AFs: 19.9

Aflatoxins in groundnuts kernels from Argentina	28-mar-24	Argentina	Poland	border rejection notification	serious	AFB1: 3.88 and 3.94; sum AFs: 5.64 and 5.77
Groundnuts China Aflatoxin	27-mar-24	China	Netherlands	border rejection notification	potentially serious	AFB1: 37; sum AFs: 58
Aflatoxin B1 in groundnuts from Paraguay	21-mar-24	Paraguay	Netherlands	information notification for attention	serious	AFB1: 9.1
Aflatoxin in Indian groundnuts	15-mar-24	India	Netherlands	border rejection notification	serious	AFB1: 11 and 5.5; sum AFs: 13 and 6.5
Aflatoxin in USA groundnuts	14-mar-24	United States	Netherlands	border rejection notification	serious	AFB1: 26 and 13; sum AFs: 30 and 15
Aflatoxin B1 in groundnuts from Paraguay	11-mar-24	Paraguay	Netherlands	information notification for attention	serious	AFB1:6.5
Aflatoxin B1 in groundnuts from Paraguay	11-mar-24	Paraguay	Netherlands	information notification for attention	serious	AFB1: 18; sum AFs: 18.9
Aflatoxin B1 in groundnuts from Paraguay	8-mar-24	Paraguay	Netherlands	information notification for attention	serious	AFB1: 2.9
Aflatoxins in groundnuts in shell from Egypt	7-mar-24	Egypt	Italy	border rejection notification	serious	AFB1: 48.6; sum AFs: 54.8
Aflatoxin in Groundnuts from China	4-mar-24	China	Netherlands	border rejection notification	potentially serious	AFB1: 14; sum AFs: 16
Aflatoxin B1 in groundnuts from Paraguay	8-feb-2024	Paraguay	Netherlands	information notification for attention	serious	AFB1: 47.7; sum AFs: 51.2
**Aflatoxins in groundnuts from Egypt**	**6-feb-2024**	**Egypt**	**Belgium**	**border rejection notification**	**serious**	**AFB1: 380; sum AFs: 590**
Aflatoxins in groundnuts from Egypt	6-feb-2025	Egypt	Belgium	border rejection notification	serious	AFB1: 310; sum AFs: 500
Aflatoxins in groundnuts from Egypt	6-feb-2026	Egypt	Belgium	border rejection notification	serious	AFB1: 230; sum AFs: 310

Aflatoxin in USA groundnuts	25-jan-24	United States	Netherlands	border rejection notification	serious	AFB1: 4.7
Aflatoxins in raw groundnuts, from the USA	19-jan-24	United States	Netherlands	alert notification	serious	AFB1: 69.6; sum AFs: 10.6
Aflatoxin in USA groundnuts	19-jan-24	United States	Netherlands	border rejection notification	serious	AFB1: not mentioned; sum AFs: 75
Groundnuts USA Aflatoxin GGB 23105780	19-jan-24	United States	Netherlands	border rejection notification	potentially serious	AFB1: 41; sum AFs: 44
Aflatoxins in groundnuts, from the USA	17-jan-24	United States	Netherlands	information notification for attention	serious	AFB1: 6.7; sum AFs: 7.8
Groundnuts Egypt aflatoxin GGB 23128479	4-jan-24	Egypt	Netherlands	border rejection notification	potentially serious	AFB1: 15; sum AFs: 15
Aflatoxins in groundnuts	3-jan-24	United States	Netherlands	information notification for attention	serious	AFB1: 4.4-8; sum AFs: 4.9-10.1
Aflatoxins in groundnuts from Egypt	3-jan-24	Egypt	Belgium	border rejection notification	serious	AFB1: 180; sum AFs: 210
Aflatoxins in groundnuts from Egypt	3-jan-24	Egypt	Belgium	border rejection notification	serious	AFB1: 94; sum AFs: 110
Aflatoxins in groundnuts from Egypt	3-jan-24	Egypt	Belgium	border rejection notification	serious	AFB1: 69; sum AFs: 90

*The only notification of this table in feed
